# Supplementary material for: Independent origin of large labyrinth size in turtles
Source: Nat Commun. 2022 Oct 11;13:5807. doi: 10.1038/s41467-022-33091-5 (PMC9553989; doi:10.1038/s41467-022-33091-5)
Supplement: Supplementary file 1 — Supplementary Information [file 41467_2022_33091_MOESM1_ESM.pdf]

Supplementary Information for

Independent origin of large labyrinth size in turtles

Serjoscha W. Evers[1,2*], Walter G. Joyce[1], Jonah N. Choiniere[3], Gabriel S. Ferreira[4,5], Christian Foth[1], Guilherme Hermanson[1,6], Hongyu Yi[7,8], Catherine M. Johnson[2], Ingmar Werneburg[4,5] & Roger B.J. Benson[2,3]

[1]Department of Geosciences, University of Fribourg, Chemin du Musée 6, 1700 Fribourg, Switzerland; [2]Department of Earth Sciences, University of Oxford, South Parks Road, Oxford OX1 3AN, United Kingdom; [3]Evolutionary Studies Institute, University of the Witwatersrand, 1 Jan Smuts Avenue, Johannesburg 2000, South Africa; [4]Senckenberg Centre for Human Evolution and Paleoenvironment an der Universität Tübingen, Sigwartstraße 10, 72076 Tübingen, Germany; [5]Fachbereich Geowissenschaften, Universität Tübingen, Hölderlinstraße 12, 72074 Tübingen, Germany; [6]Laboratório de Paleontologia de Ribeirão Preto, FFCLRP, Universidade de São Paulo, Ribeirão Preto, Brazil; [7]Key Laboratory of Vertebrate Evolution and Human Origins of Chinese Academy of Sciences, Institute of Vertebrate Paleontology and Paleoanthropology, Chinese Academy of Sciences Beijing 100049, China; [8]CAS Center for Excellence in Life and Paleoenvironment Beijing 100044, China

*Serjoscha W. Evers. Email: serjoscha.evers@unifr.ch

This PDF file includes:

Supplementary methods
- **1 CT and 3D data deposition**
- **2 Segmentation of 3D models**
- **3 Reconstructed specimens**
- **4 Landmark concept**
- **5 Variables for hypothesis testing**
  - **5.1 Cranial box volumes**
  - **5.2 Braincase aspect ratio**
  - **5.3 Relative neck lengths**
  - **5.4 Neck retraction ability and type**
  - **5.5 Habitat ecology of extant and fossil taxa**
  - **5.6 Habitual habitat**
  - **5.7 Forelimb webbing**
- **6 Composite tree and time calibration**
- **7 Additional notes on analyses**
- **8 Institutional abbreviations**
- **9 Legends for supplementary datasets 1–20**

Supplementary notes
- **1 Sensitivity tests for phylogenetic comparative analyses**
- **2 Short additional notes to PCA analysis**
- **3 Labyrinth shape regressions excluding marine taxa and/or chelonioids**
- **4 Labyrinth shape regressions excluding landmarks from the ASC loop**

Supplementary Figures

Supplementary Tables

Supplementary References

Supplementary methods

1 CT and 3D data deposition

To generate 3D model data for endosseous labyrinth and crania, we used high-resolution micro-computed tomography (CT) scans (for 167 specimens) and magnetic resonance imaging (MRI) scans (for 1 specimen). The tomographic datasets were largely collected by our team, but some scans were passed on to us from other researchers, or downloaded from online repositories or data supplements of published papers. We deposited all CT scans collected by us in the online repository MorphoSource (www.morphosource.org/), and the majority of scans not produced by us are also available online on MorphoSource or different repositories (Supplementary Data 1). Some museums do not currently allow the public deposition of CT scans and require that the CT data are stored and curated by the museum, so that some CT scans are only available upon request with the respective institution. Details for each scan and where to find it are listed in Supplementary Data 1. Scanning facilities, scanning parameters, and scanning procedures varied for each specimen, and respective information is provided alongside the CT slice data and derivative models in their respective repository.

Within the MorphoSource repository, CT scans can be curated either by the uploader, or by the institution to which the respective specimen belongs, and access for many scans and digital specimens is subjective to curatorial permission online. All scans of specimens that come from museums without download-restrictive policies were uploaded by us for direct download (i.e. not requiring download permission), others were uploaded with respective restrictions implemented.

We also uploaded 3D model data of endosseous labyrinths that were used for landmarking as well as cranial models that were used to obtain cranial measurements to MorphoSource. Links to all 3D labyrinth models can be found in Supplementary Data 1. For those specimens for which tomographic data is also deposited on MorphoSource, model and tomographic data are cross-linked. This is possible because each file within MorphoSource has a unique Media ID, and tomographic media were selected as parent media for respective models. For specimens for which CT data are not on MorphoSource, model data were uploaded without cross-linked parent tomographic media. Thus, all model data for this project are in one place, organized in a MorphoSource Media Collection 000372533 called “Evers. 2021. Extant and fossil amniote labyrinth and cranial models” and accessible here: www.morphosource.org/projects/000372533.

2 Segmentation of 3D models

3D models were generated by manual segmentation in Materialise Mimics 18.0–19.0. Specimen-specific thresholds were used, and some specimens, specifically but not exclusively fossils, were segmented without applying thresholds by using the manual mask addition tool, for instance when threshold difference between the semicircular canal walls and infilled rock matrix was low. We only segmented one endosseous labyrinth for each specimen, preferentially from the left side but the right side was chosen when the skull was damaged on the left side. In addition to labyrinth models, models of the cranium were produced to be used for specimen-specific cranial measurements. For some fossils, matrix surrounding the crania was digitally removed to enable landmarking. All resulting models were exported as PLY files, and labyrinth and cranial models from the same specimen preserve their special relationships with one another as well as their digital scale.

3 Reconstructed specimens

For some fossil specimens, the cranium or labyrinth was taphonomically distorted or broken, and digital reconstruction was applied to include data of the respective specimens into our study. Reconstructions were done by producing two or more 3D models in Mimics or Avizo, which were then re-articulated in Blender. We exported the reconstructed models in the PLY format, keeping the original model sizes and geometric relationships between labyrinths and crania. Reconstruction details given below are also stored with the respective 3D models in MorphoSource.

Adocus lineolatus. Within the braincase of *Adocus lineolatus* (CMM 60-15), the ventral process of the left supraoccipital was broken off the remainder of the supraoccipital (which is preserved in articulation). The ventral process of the supraoccipital houses the common crus area of the endosseous labyrinth, and therefore the supraoccipital breakage affected the endosseous labyrinth model. To reconstruct the endosseous labyrinth, we produced two separate models in Mimics, for the prootic-opisthotic part of the labyrinth, and the supraoccipital part, respectively. We imported both models into Blender, and digitally re-articulated them.

Annemys sp. In *Annemys* sp. (IVPP V18106), the skull roof is broken off the rest of the cranium, and both parts are separated by a large fracture line (see also Brinkman et al.[1]). We segmented both parts separately in Mimics, and re-articulated the skull roof to the rest of the skull in Blender.

Basilemys gaffneyi. DMNH 130896, a cranium of *Basilemys gaffneyi*, is fractured into numerous parts that are, for the most part, preserved in very good articulation. However, the endosseous labyrinth was affected by fractures. The ventral part of the right prootic was affected by a fracture, resulting in a collapse of the dorsal parts of the prootic, which houses the anterior semicircular canal (ASC). As a result, the ASC was ventrally deflected at the prootic-supraoccipital suture, but the canal curvature and length were still perfectly preserved within the displaced prootic. Therefore, we separated the ASC from the remainder of the endosseous labyrinth as a separate model, and re-articulated both parts in Blender.

Dirqadim schaefferi. In our scanned specimen of *Dirqadim schaefferi* (AMNH 30038)[2], the ventral parts of the supraoccipital have collapsed within the otic capsule, so that the common crus part of the endosseous labyrinth was displaced with regard to the labyrinth parts housed within the prootic and opisthotic. The segmented model of the crushed ear was imported in Avizo, and we separated the supraoccipital part of the ear via manual 3D manipulation. The supraoccipital part and remainder of the endosseous labyrinth were then exported as separate models, and digitally re-assembled in Blender.

Kayentachelys aprix. The specimen TMM 43670-2 of *Kayentachelys aprix* is generally very three-dimensionally preserved (see also Gaffney & Jenkins[3]). However, on the left side of the specimen, parts of the infraorbital bar, corresponding to the posterior half of the maxilla and most of the jugal, are broken away from their original position and preserved in a slightly misplaced position. This broken part was digitally cut away from the remainder of the skull along the naturally occurring breaks, using Avizo, and both skull parts were exported as separate models. Those were then digitally re-assembled in Blender.

Latentemys plowdeni. The specimen NHMUK R11998 of *Latentemys plowdeni* is preserved in two parts, whereby on includes the basicranium and parts of the skull roof, and the other most of the snout and the palate. Both parts of the skull fit perfectly together along the skull roof, whereas

some gaps remain along the palate when both parts are articulated (see Gaffney et al.[4]). Both parts were CT-scanned separately, and the segmented models were digitally articulated in Blender.

4 Landmarking concept using midline skeletons on reconstructed semicircular duct trajectories

Due to the intersection of the posterior part of the lateral semicircular duct and the ventral parts of the posterior semicircular duct within the endosseous labyrinth cavity of turtles, endosseous labyrinth models of turtles provide only a rough approximation of the shape of the underlying tissue organ. As it is the endolymphatic flowpath within this duct-tissue system that determines function, we used the recommendations by Evers et al.[5] to reconstruct semicircular duct trajectories from 3D endosseous labyrinth models, guided by comparisons with actual membranous labyrinths of turtles. Reconstructed semicircular models were then skeletonized and landmarked. This reconstruction methods has also recently been used by some of us in a comparative analysis of archosaur labyrinths[6].

To arrive at midline skeletons, the three semicircular canals needed to be isolated from their original endosseous model. This model modification, as well as skeletonization and landmarking was done in Avizo lite 9.2. Models of the endosseous labyrinths and crania were imported as PLY files into Avizo. We cut the endosseous labyrinths models using 3D mask-editing in the segmentation window of Avizo to isolate the anterior, posterior, and lateral semicircular canals. We isolated the semicircular canals by extending the posterior and lateral semicircular canals through the secondary common crus. Hereby, the lateral semicircular canal was posteriorly extended through the secondary common crus in the same horizontal plane as the exposed anterior portion of this canal (see Evers et al.[5]). In some turtle species, the posterior part of the semicircular duct also leaves a partial impression in the secondary common crus, further aiding the accurate reconstruction of the full length of the lateral semicircular canal. The visible portion of the posterior semicircular canal was ventrally extended to curve laterally around the lateral semicircular canal, as predicted by membranous duct morphology. In order to do this, the position of the posterior ampulla had to be approximated, and we followed the protocol of Evers et al.[5] for this reconstruction. Our canal reconstructions are taken to be closer approximations of the underlying soft tissue anatomy than unmodified models of the endosseous cavities housing this organ. Because midline skeletons produced in Avizo terminate slightly prior to the end of segmented canals, we extended our canal models on either side to achieve sufficiently long skeletons (i.e., the skeletons extend beyond the inferred position of the ampullae and canal-common crus intersections). Isolated semicircular canals were skeletonized using the *autoskeleton* function in Avizo with a 'smooth' coefficient of 0.5, and 'attach to data' parameter values of 0.5 to create streamlined skeletons without artifacts.

Midline skeletons of the semicircular canals were landmarked using six conventional fixed landmarks, which describe the starting points of canals at their intersection with their respective ampulla, and the endpoints of canals at their intersection with the common crus (see Supplementary Fig. 1i). For each semicircular canal, an open semilandmark curve was placed on the midline skeleton between the start and endpoint landmarks (i.e. from ampullae to common crus). Additional to the landmarks on the midline skeleton, a closed semilandmark loop was placed around the inner perimeter of the ASC (Supplementary Fig. 1h), whereby the landmarks were placed as extending laterally from the dorsal arch of the ASC, continuing ventrally around

the canal and dorsally up the common crus to close the loop. During landmarking, each semilandmark loop was sampled with an arbitrary number of semilandmarks, densely packed around the curvature of the loop. At the analytical stage, semilandmarks within each loop were re-sampled to the mean number of semilandmarks used across specimens, so that each specimen had the same number of semilandmarks.

We place a further semilandmark loop around the cross-section of the ASC near its ampulla, defining the circumference of the ASC. These landmarks were not used for geometric morphometric analyses, and only used to calculate ASC circumferences as the sum of distances between landmark positions. This data is not further used here, but will be explored elsewhere.

5 Variables for hypothesis testing

We determined several size-related, behavioural, ecological, and taxonomic variables to be used in regression analyses/statistical hypothesis testing. All variable scorings for all taxa are summarized in Supplementary Data 2, and the different variables are discussed below.

5.1 Cranial box volumes

Cranial box volumes are the smallest virtual cube to contain the full cranium of a specimen, and were measured as a proxy of skull size for each complete or near-complete specimen by multiplying the maximum anteroposterior length (between the premaxilla and occipital condyle) with the maximum dorsoventral height (between the skull roof and the ventral surface of the basicranium) and maximum mediolateral width (between the temporal skull bones, usually near the antrum postoticum perimeter). Measurements were taken as straight-line measurements in Mimics, either on slices directly, or on the 3D model in the model viewing panel of Mimics. For specimens with minor damage to the respective areas, measurements were approximated when possible. However, for specimens in which the preservation does not allow approximation of one of the measurements (for instance, when only the basicranium is preserved), no box volume parameters were measured. Linear measurements and box volumes for all specimens are included in Supplementary Data 2.

5.2 Braincase aspect ratio

To test spatial constraint hypotheses in our model tests, we computed a braincase aspect ratio variable by dividing skull height by skull width, following previous authors such as Bronzati et al.[6]. This was done directly in R, so that the respective ratios are not part of Supplementary Data 2.

5.3 Relative neck lengths

Turtle head movements are facilitated by their necks, and neck length and mobility are important for different head movement behaviours (e.g. refs.[7-8]). We established relative neck length categories by contrasting absolute neck length with absolute carapacial length, following the procedure of Joyce et al.[8]. We calculated the percentage proportion of neck length with regard to

carapacial length, and created relative neck length categories according to those percentages, with a neck-to-carapace proportion of >70% being categorized as ‘extreme’; proportions of 50–69% as ‘long’; proportions of 35–49% as ‘intermediate’; and proportions of <35% as ‘short’. The length of the carapace was measured from the anterior margin of the nuchal to the posterior margin of the pygal, or topological equivalents in turtles that lack a pygal. Absolute neck length was measured as the cumulative length of the centra of all eight cervical vertebrae. Measurements were either taken on 3D models in Mimics (for specimens for which we had full body scans available), or on osteological pictures in ImageJ, using the ‘set scale’ function in that software to calibrate measurements against the scale bars included in photographs. The same method was used on published images of turtle specimens that preserve the neck and carapace for taxa for which we did not have photographs. Although neck and carapace were always taken from a single individual, these individuals were other individuals than the ones we used for generating the endosseous labyrinth models, with the exception of those specimens for which we had full body scans. Details of specimen identification, absolute neck and carapace lengths, as well as resulting ratios are summarized in Supplementary Table 1, and ratios are also listed in Supplementary Data 2.

5.4 Neck retraction ability and type

Extant turtles are generally able to retract their necks underneath the carapace. Neck retraction is facilitated by cervical vertebra anatomy[9–10], but the two major lineages of crown turtles, pleurodires and cryptodires, have evolved different neck retraction mechanisms. In cryptodires, necks are retracted along a vertical plane and the neck if folded in a sigmoidal fashion to hide the skull underneath the carapace (hence, “hidden-necked turtles”). In pleurodires, the principal plane for neck tucking is horizontal (hence, “side-necked turtles). It is unclear when exactly turtles evolved neck retraction, but studies have argued that early stem-turtle (including *Proganochelys quenstedtii*), meiolaniforms (including *Meiolania platyceps*), helochelydrids (incl. *Naomichelys speciosa*), paracryptodires, as well as thalassochelydians had only an incomplete neck retraction ability in which most of the head would remain exposed (e.g., refs.[10–12]). For all stem turtles, cervical vertebra anatomy also does not suggest that either the cryptodiran nor pleurodiran mechanism was developed. The evolution of full neck retraction has been proposed as important for labyrinth size evolution[13], but this hypothesis has so far not been tested.

We coded two morphofunctional variable related to head retraction. The first variable, neck retraction ability, differentiates between turtles that incompletely or fully retract their head underneath the shell. Besides all stem turtles, several macrocephalic crown turtles which have lost the ability to withdraw the head fully (e.g., ref.[14]) were coded as incomplete: chelonioids, *Platysternon megacephalum*, *Macrochelys temminckii*, *Phosphatochelys tedfordi*, *Ummulisani rutgersensis*, *Peltocephalus dumerilianus*. As a second variable, we recorded the principal plane for head movements during neck retraction (none or vertical or horizontal). For this variable, cryptodires or pleurodires without the ability to (fully) retract their necks are scored according to their phylogenetic expectation as vertical (cryptodires) of horizontal (pleurodires), as these turtles still share general cervical vertebral features of their respective clades, but neck retraction is prohibited by their large head sizes. Neck classifications for all species are listed in Supplementary Data 2.

5.5 Habitat ecology of extant and fossil taxa

Three general habitat ecology distinctions were made: 'marine', 'freshwater', 'terrestrial'. Although many continental turtles are somewhat amphibious, resulting in a difficult distinction between 'freshwater' and 'terrestrial', turtle species were herein scored according to their predominant habitat preference in which they forage: all extant chelonioids were scored as 'marine'; all extant pleurodires, trionychians, kinosternids, chelydrids were scored as 'freshwater'; all extant testudinids were scored as 'terrestrial'. Geoemydids and emydids were generally scored as 'freshwater', with the exception of the following species, which were scored as 'terrestrial': *Cuora flavomarginata*, *Cuora mouhotii*, *Cyclemys dentata*, *Geoemyda spengleri*, *Rhinoclemmys pulcherrima*, *Glyptemys insculpta*, *Terrapene carolina*, *Terrapene ornata*. Ecological classifications for each species are listed in Supplementary Data 2.

The ecology of fossil turtles was assessed on a taxon-by-taxon basis. Categorization was assessed with reference to the available published literature about the ecology of extinct fossils. We classified all fossil chelonioid turtles, including protostegids, as marine. This supported by the depositional environments as well as marine adaptations for all of the species considered in our study (see Evers & Benson[15] for comments). For pleurodires, we followed the Ferreira et al.[16], Gaffney et al.[2], and Gaffney et al.[4] to distinguish between marine and freshwater pelomedusoids. The following fossil turtles were scored as 'terrestrial': the early stem turtles *Proganochelys quenstedtii*, *Australochelys africanus* and *Kayentachelys aprix* based on depositional environments and postcranial anatomy (e.g., refs.[17–20]); the meiolaniform *Meiolania platyceps* based on postcranial anatomy including armour, shell histology and general habitat preferences of the group[20–23]; the stem trionychian *Basilemys* sp. based on the presence of extensive limb armour, foot anatomy, skeletal robusticity, and cranial adaptations to high-fiber herbivory (e.g., ref.[24]); the testudinoid *Stylemys nebrascensis* based on general anatomical considerations and depositional environment[25]). Ecological classifications for all fossil taxa are listed in Supplementary Data 2.

5.6 Habitual habitat

Although turtle habitats can grossly be divided into marine, freshwater aquatic, and terrestrial categories, extant turtle species show different behaviours within their preferred environments. Therefore, we classified whether extant turtles show extensive burrowing behavior, are terrestrial walkers, aquatic bottom dwelling species, or open water swimmers. Open water swimmers can be marine or non-marine aquatic. Classifications for each species are listed in Supplementary Data 2.

5.7 Forelimb webbing

The limbs of turtles are highly specialized to their environments. This is evident from ratio measurements of individual forelimb parts[19], but also from the extent of the webbing between finger digits. Forelimb element ratios distinguish terrestrial turtles from semiaquatic ones, and semiaquatic turtles from marine turtles. However, the degree of hand webbing shows further nuances in semiaquatic turtles, so that we decided to use webbing as a proxy for 'aquaticness', and therefore habitat ecology for extant turtles of our sample. Our forelimb webbing categories

are the same as proposed by Foth et al.[26], expanded to our taxon sample, and are listed for each species in Supplementary Data 2.

6 Composite tree and time calibration

To perform phylogenetic comparative methods on the full dataset including fossils, we required a phylogenetic tree that includes all focal taxa of our sampling. As no single published 'conventical' phylogeny existed that fulfilled this criterion, we constructed a composite tree that includes all of the taxa for which we have labyrinth data by informally combining topologies from several published phylogenies. We used the tree topology of Pereira et al.[27], which is a time-calibrated molecular phylogeny of extant turtles based on 13 loci for 294 living turtle species, as a constraint on the relationships of extant turtles. Fossil pleurodires were added to the tree following the topology of Ferreira et al.[16], using the analysis from that paper which included a molecular backbone constraint for extant pleurodires. On the turtle stem lineage, the positions of basal stem-turtles (i.e., *Australochelys africanus*, *Kayentachelys aprix*, *Eileanchelys waldmani*), sinemydids (represented by *Ordosemys* sp.), xinjiangchelyids, thalassochelydians, and sandownids follow Evers & Benson[15]. The ingroup relationships of plesiochelyids follows Evers et al.[28], but *Solnhofia parsonsi* was constrained to be the sister taxon of *Sandownia harrisi*, following Evers and Joyce[29] and Joyce et al.[30]. Paracryptodire relationships follow Lyson & Joyce[31], but *Naomichelys speciosa* was additionally constrained as the earliest branching paracryptodire, following unpublished phylogenetic results of some of us (SWE, WGJ), which support recent comparative anatomical considerations[32–33]. *Kallokibotion bajazidi* was constrained as a meiolaniform, following Sterli et al.[34]. *Adocus lineolatus* and *Basilemys gaffneyi* were placed as sister taxa to each other on the stem of Trionychia, following most recent phylogenies that include *Adocidae* and *Nanhsiungchelyidae* as a monophyletic clade[25–39] of stem-trionychians[15,35,39]. Within Trionychia, *Allaeochelys libyca* was treated as the sister to *Carettochelys insculpta*[15], and *Petrochelys kyrgyzensis* was constrained to be a stem-trionychid, following the implied weighting topology published by Brinkman et al.[40]. *Axestemys infernalis* was included as the sister taxon to the *Apalone* group, following Vitek[41]. *Stylemys nebrascensis* was placed as the sister group to the *Gopherus* group within testudinids. This placement follows biogeographic considerations and the traditional hypothesis that all North American testudinids share a more recent common ancestor than other testudinids (e.g., ref.[42]). However, in recent phylogenetic analyses, the position of *Stylemys nebrascensis* is not stable, and a relationship with gopher tortoises has often not been supported[43–44]. Protostegids were placed as stem-chelonioids, following Raselli[45] and Evers et al.[28]. Non-protostegid chelonioid relationships are: the Eocene sea turtles *Argillochelys antiqua*, *Eochelone brabantica*, and *Puppigerus camperi* were included as stem-cheloniids (see also Evers et al.[28]); *Nichollsemys baieri* was constrained as a stem-chelonioid in a more crownward position that protostegids; and *Allopleuron hofmanni* was included as the sister taxon of *Dermochelys coriacea*.

The phylogeny including fossils was primarily used for the evolutionary analysis of labyrinth size. Key results from this analysis (ancestrally small labyrinth size for turtles that increases from the node joining *Eileanchelys waldmani* and more crownward turtles; secondary size reduction in testudinids) are not affected by contentious parts of our phylogeny, which primarily concerns the placements of several crownward stem-turtle clades (e.g. sinemydids) alternatively as stem-cryptodires, and the placement of the stem-chelonioid protostegids alternatively as closely related to Jurassic thalassochelydians. Thus, we have not explored the effect of alternative phylogenetic topologies on our analyses.

For the time-calibration of our composite tree, we used two different *a posteriori* methods: the stochastic *cal3* method of Bapst[46], and the minimum branch length (mbl) method[47], by using commands from the paleotree[48], strap[49], Claddis[50] and ape[51] packages. Both methods use temporal ranges of taxa, which were compiled from the literature (Supplementary Data 6), to calibrate internal nodes. As the primary source phylogenies[16,27] already included calibrations from most internal nodes, we only calibrated nodes that resulted from the inclusion of fossil taxa not originally included in any of the source phylogenies. For the cal3 calibration, we extended the calibration procedure to also include deep nodes of the phylogeny (Testudines, Cryptodira, Pleurodira, Trionychia, Pelomedusoides), as these have very deep ages in the data of Pereira et al.[27], resulting in conflicts with the fossil record. As the cal3 method is a stochastic calibration, we ran 100 calibrations, from which one calibrated tree was chosen at random for our comparative phylogenetic analyses. To assess the impact of different time-calibrations, we performed comparative phylogenetic analyses both on one randomly-sampled cal3 tree (Supplementary Data 7 & Supplementary Figure 2) and the mbl tree Supplementary Data 8 & Supplementary Figure 3). These two calibrated trees resulted in very similar analytical results (see below), indicating that our results are not fundamentally influenced by different time calibrations.

7 Additional notes on analyses

The landmark data and R scripts used for analyses in this paper are provided at Zenodo (doi.org/10.5281/zenodo.7024572). All specimen data and explanatory variables are provided in Supplementary Data 2. Supplementary Data 3 contains the landmarks data for all specimens as individual csv-files. Supplementary Data 4 contains the information for sliding semilandmarks, and Supplementary Data 5 contains landmark colour information used to generate deformation plots with the PCA analyses. Supplementary Data 6 contains the age data required for phylogenetic time-calibrations, whereas Supplementary Data 7 and 8 are the cal3 and mbl calibrated phylogenetic trees, respectively, each as a text-file with nexus syntax. Our turtle specific analyses can be repeated by executing the scripts provides in Supplementary Data 10–13 & 16, whereby Supplementary Data 10 performs the PCA analysis, Supplementary Data 11 performs the labyrinth shape regression analyses; Supplementary Data 12 performs the size-corrected PCA and regression analysis; Dataset 30 performs the labyrinth size regression analyses, and Supplementary Data 16 performs the ancestral state reconstruction. Supplementary Data 14 & 15 each contain a table listing all model comparisons we ran for the labyrinth size regression analyses for both the cal3 and mbl trees, respectively, and are thus outputs of Supplementary Data 13. Supplementary Data 17 contains the specimen information and skull measurements of our amniote data. Supplementary Data 18 contains the landmark data for amniotes as individual csv files, and Supplementary Data 19 contains the sliding semilandmark information for the amniote GPA analysis. Supplementary Data 20 is the script to read this data, run a joint GPA analysis and to produce Figure 4 of the main text. Supplementary Data 21 contains the source file for all main text and supplementary figures that are based on graphs.

8 Institutional Abbreviations

AMNH - American Museum of Natural History, New York City, USA; **BP** - Evolutionary Studies Institute (formerly Bernhard Price Institute for Palaeontological Research), University of the

Witwatersrand, Johannesburg, South Africa; **BSPG** - Bayerischa Staatssammlung für Paläontologie und Geologies, Munich, Germany; **CAMSM** - Sedgwick Museum, University of Cambridge, UK; **CMM** - Carter County Museum, Ekalaka, USA; **CRI** - Chelonian Research Institute, Oviedo, USA; **CSIP** - Ceteacean Strandings Investigation Programme, UK; **DMNH** - Denver Museum of Nature & Science, Denver, USA; **FHSM** - Fort Hays State University, Sternberg Museum of Natural History, Hays, USA; **FMNH** - Field Museum of Natural History, Chicago, USA; **GPIT** - Geologisch-Paläontologisches Institut der Universität Tübingen, Tübingen, Germany; **IRSNB** - Institut Royal des Sciences Naturelles des Belgique, Brussels, Belgium; **IVPP** - Institute of Vertebrate Paleontology and Paleoanthropology, Beijing, China; **JM -** Jura Museum, Eichstätt, Germany; **KUVP** - University of Kansas Biodiversity Institute, Lawrence, USA; **LIRP** - Laboratório de Ictiologia de Ribeirão Preto, University of São Paulo, Brazil; **MB** - Museum für Naturkunde Berlin, Berlin, Germany; **MDET -** Musée des Dinosaures, Espéranza, France; **MIWG** - Museum of the Isle of Wight, Sandown, UK; **MJSN** - Jurassica Museum, Porrentruy, Switzerland; **MNHN** - Muséum National d'Histoire Naturelle, Paris, France; **MPMA** - Museu de Paleontologia de Monte Alto, Monte Alto, Brazil; **MS** - MorphoSource Specimen ID, Duke University, Durham, USA; **NCSM** - North Carolina Museum of Natural History, Raleigh, USA; **NHMUK** - Natural History Museum, London, UK; **NMB** - Naturhistorisches Museum Basel, Basel, Switzerland; **NMS** - National Museum of Scotland, Edinburgh, Scotland; **OUMNH** - Oxford University Museum of Natural History, Oxford, UK; **OUVC** - Ohio University Vertebrate Collections, Athens, USA; **PCHP** - Chelonian Research Institute/Peter C.H. Pritchard, Oviedo, USA; **PIMUZ** - Paläontologisches Institut der Universität Zürich, Zurich, Switzerland; **QM** - Queensland Museum, Brisbane, Australia; **SAM** - South Australian Museum, Adalaide, Australia; **SMF** - Forschungsinstitut und Naturmuseum Senckenberg, Frankfurt am Main, Germany; **SMNS** - Staatliches Museum für Naturkunde Stuttgart, Stuttgart, Germany; **TM** - Teylers Museums, Haarlem, The Netherlands; **TMM** - Texas Memorial Museum, Austin, USA; **TMP -** Royal Tyrrell Museum, Drumheller, Canada; **UA** - University of Antananarivo, Antananarivo, Madagascar; **UCMP** - University of California Museum of Paleontology, Berkeley, USA; **UCMVZ** - University of California Museum of Vertebrate Zoology, Berkeley, USA; **UF** - Florida Museum of Natural History, Gainesville, USA; **UFR** - University of Fribourg, Fribourg, Switzerland; **UMMZ** - University of Michigan Museum of Zoology, Ann Arbor, USA; **UMZC** - University of Cambridge Museum of Zoology, Cambridge, UK; **USNM** - United States National Museum, Washington D.C., USA; **WAM -** Western Autralian Museum, Perth, Australia; **YPM** - Yale Peabody Museum, New Haven, USA; **ZIN -** Zoological Institute of the Russian Academy of Sciences, St Petersburg, Russia

9 Legends for Datasets 1–20

These datasets are published in Zenodo (doi.org/10.5281/zenodo.7024572).

Data 1. Spreadsheet including specimen data, and MorphoSource links to 3D models for each specimen. The CT data are linked to each 3D model within MorphoSource. For datasets with restricted download policies implemented by the museums in charge of the specimens, contact details as listed in MorphoSource are provided.

Data 2. Spreadsheet including specimen data, explanatory variables and measurements for turtle labyrinth analyses. This is read by scripts provided as Datasets 10–13 & 16.

Data 3. Collection of turtle landmark data, as individual csv files. This is read by scripts provided as Datasets 10–30 & 16.

Data 4. Csv file containing information about sliding semilandmarks (for GPA analysis). This is read by scripts provided as Datasets 10–13 & 16.

Data 5. Csv file containing colour codes for landmarks, used for deformation plots along PC axes. This is read by scripts provided as Datasets 10–13 & 16.

Data 6. Csv file containing age data for fossil turtle species, alongside information about the fossil prevenance and museum staff responsible for curation and/or collection management..

Data 7. Text file containing cal3-calibrated phylogenetic tree in nexus syntax. This is read by scripts provided as Datasets 10–13 & 16.

Data 8. Text file containing mbl-calibrated phylogenetic tree in nexus syntax. This is read by scripts provided as Datasets 10–30 & 16.

Data 9. Text file with R script to load landmark data, variable data, phylogenetic data from Datasets 2–5 & 7–8 and to 2B-PLS analysis for te verification of our landmarking scheme.

Data 10. Text file with R script to load landmark data, variable data, phylogenetic data from Datasets 2–5 &7–8 and to perform GPA and PCA analysis. Script creates Figure 1 from main text.

Data 11. Text file with R script to load landmark data, variable data, phylogenetic data from Datasets 2–5 & 7–8 and to perform GPA analysis and labyrinth shape regressions.

Data 12. Text file with R script to perform size- and braincase aspect ratio-corrected PCA and regression analyses, and to get deformation plots. Script creates Figure 2 from main text.

Data 13. Text file with R script to load landmark data, variable data, phylogenetic data from Datasets 2–5 & 7–8 and to perform GPA analysis and labyrinth size regressions and model comparisons.

Data 14. Spreadsheet showing full list of models tested in turtle labyrinth size regressions and model comparison data (AICc, etc.) for analysis runs based on cal3-calibrated phylogenetic tree.

Data 15. Spreadsheet showing full list of models tested in turtle labyrinth size regressions and model comparison data (AICc, etc.) for analysis runs based on mbl-calibrated phylogenetic tree.

Data 16. Text file with R script to load landmark data, variable data, phylogenetic data from Datasets 2–5 & 7–8 and to perform GPA analysis, labyrinth size regression analysis used for Figure 3 in main text, and ancestral state reconstructions used also in Figure 3.

Data 17. Spreadsheet including specimen data and cranial measurements for amniote labyrinth GPA and labyrinth size plot. This is read by the script provided as Dataset 20.

Data 18. Collection of turtle landmark data, as individual csv files. This is the data file for script provided as Dataset 20.

Data 19. Csv file containing information about sliding semilandmarks (for GPA analysis). This is read by the script provided as Dataset 20.

Data 20. Text file with R script to load amniote landmark data, amniote measurements, and sliders from Datasets 17–19 and to perform GPA analysis and plots shown used as Figure 4 of main text.

Supplementary notes

1 Sensitivity tests for phylogenetic comparative analyses using the mbl-tree

Our Procrustes distance shape regressions are only minorly affected by using a differently calibrated tree. Using the minimum branch length tree results in nearly the same relative importance of variables and models, and R^2 values and significance levels for variables are nearly identical (compare Table 1 from main text with Supplementary Table 2). As for the analysis presented in the main text, the best model tested according to R^2 and in which all individual variables are significant takes the forms of labyrinth shape ~ skull box volume * braincase aspect ratio + labyrinth centroid size. As with the cal3-tree-based analyses presented in the main text, ecological habitat variables and morphofunctional neck variables are insignificant when included in bivariate or multiple regression analyses.

Our pGLS regressions on labyrinth centroid size using the alternative mbl-calibrated tree also show that tree calibration has no major effect on our analyses or their interpretations. Model comparison of the mbl-based analyses retrieves ten models with non-negligible AICc values, all of which are among the set of 13 models with non-negligible AICc in the main-text analysis, which uses a cal3-calibrated tree (compare Table 2 from main text with Supplementary Table 3). The sequence of models (i.e. from best getting AICc-worse) is not identical, but very similar, and models with non-negligible AICc values should all be considered anyway. The same set of variables is returned as part of the non-negligible models, and models have near identical R^2. Thus, the analysis using the mbl tree supports the results presented based on the cal3 tree in the main text.

2 Short additional notes to PCA analysis

Although we comment on the position of specific species and higher clades in the morphospace of our PCA analysis or landmark data, we only show a version of the morphospace in which specimens are colour coded according to habitat ecology. Supplementary Figure 4 additionally shows a taxonomic colour coding of specimens, and in addition, specimens are identified via numbers plotted onto their point symbols. Supplementary figures 5–8 show deformations of the labyrinth landmark configurations at extreme points of PC axes 1–6. Disparity analysis and further comments on the taxonomic distribution of specimens in the morphospace will be provided elsewhere.

3 Labyrinth shape regressions excluding marine taxa and/or chelonioids

Among our sampled turtles, chelonioid sea turtles have unusually derived vestibular morphology, with unusually high aspect ratios (i.e., dorsoventrally tall and anteroposteriorly short labyrinths)

and unusually thick semicircular canals. To test if any of our detected patterns, particularly the absence of independently significant effects of ecological variables, may be influenced by the unusual labyrinth shape of sea turtles, we performed two additional procD.pgls analyses that excluded marine species (i.e., chelonioid sea turtles, thalassochelydians, marine pleurodires; N = 109) or excluded only chelonioids (N = 123). This was implemented based on a reviewer's comment.

When only chelonioid sea turtles are excluded, the best model (i.e. including only significant terms, and simultaneously maximising R^2) takes the same form as the best model reported in the main text (labyrinth shape ~ skull box volume*braincase aspect ratio + labyrinth centroid size; Supplementary Table 4). The ecological effects 'terrestrial', 'freshwater', and 'marine.all', as well as the functional neck parameters are not included in this model, and also do not have significant relationships with labyrinth shape whem analysed individually in bivariate regressions (Supplementary Table 4) and most multivariate regressions. The only exception to this is 'terrestriality', which becomes marginally significant ($p = 0.045$) when included in a model with skull box volume, braincase aspect ratio and labyrinth centroid size. But this is redundant with the strong, and consistently significant effect of the interaction term between skull box volume and braincase aspect ratio, which themselves are significant and well-supported (Supplementary Table 4). 'Terrestriality' becomes non-significant when included alongside an interaction term between skull box volume and braincase aspect ratio in multiple regressions (Supplementary Table 4). Thus, our results from the full analysis, and particularly the absence of significant ecological effects or morphofunctional effects related to the neck on explaining turtle labyrinth shape variation are upheld when chelonioids are excluded.

When all marine taxa are excluded (i.e., chelonioids, bothremydids, stereogyines, thalassochelydians), the best model (i.e., including only significant terms, and simultaneously maximising R^2) is still identical to that of the main analysis including all taxa (labyrinth shape ~ skull box volume *braincase aspect ratio + labyrinth centroid size). This strongly supports that our results and interpretations presented in the main text are not driven by the inclusion of marine taxa.

Nevertheless, the effect of excluding all marine taxa can be seen when comparing the results of bivariate regression with those from the full analysis using all taxa. For example, most allometry-related variables are only significant when included alongside the braincase aspect ratio variable, except for skull height, which remains significant even in a bivarite regression ($p = 0.033$) (Supplementary Table 5). This differs from the analysis using the full taxonomic dataset, in which all allometry-related variables are highly significant on their own. The braincase aspect ratio is significant in bivariate models using all datasets. The interaction term between skull box volume and braincase aspect ratio is also significant when marine turtles are excluded (Supplementary Table 5), as in the full analysis. Morphofunctional neck variable are always non-significant (Supplementary Table 5), as in the full analysis. When analyzed by themselves, the ecological variables 'terrestrial' and 'freshwater' are significant when excluding marine turtles (Supplementary Table 5), whereas they were not using the full dataset. However, their effects are redundant with the allometric effects in more complex multivariate models (Supplementary Table 5). Thus, although the results from the analysis excluding all marine turtles differs more strongly from the main analysis including all taxa than the one only excluding chelonioids, it also supports the hypothesis that ecology has no independently significant effect in explaining turtle labyrinth shape variation.

4 Labyrinth shape regressions excluding landmarks from the ASC loop

We performed an additional labyrinth shape regression based on a reviewer's comment, which suggested to test if the ASC inner loop has a specific influence on our analyses, as the loop ultimately captures variation related to the thickness of the semicircular canal, but also variation of the saccule, vestibule and ampulla. In order to perform this text, we excluded the ASC landmarks from the dataset and ran the procD.pgls regression (*N*=138 taxa, including fossils) with the same ecological and functional parameters as the analysis presented in the main text. The results suggest the same conclusions, as the best model (including only significant terms, and simultaneously maximising R^2) is identical to that of the main analysis using all landmarks (i.e., labyrinth shape ~ skull box volume *braincase aspect ratio + labyrinth centroid size). As in the main analysis, neither the morphofunctional neck variables, not the ecological variables analyzed are significantly related to labyrinth shape (Supplementary Table 6). Instead, the braincase aspect ratio variable as well as allometric variables receive significant support in bivariate and multivariate analyses (Supplementary Table 6), similar to our main text analyses.

Supplementary Figures

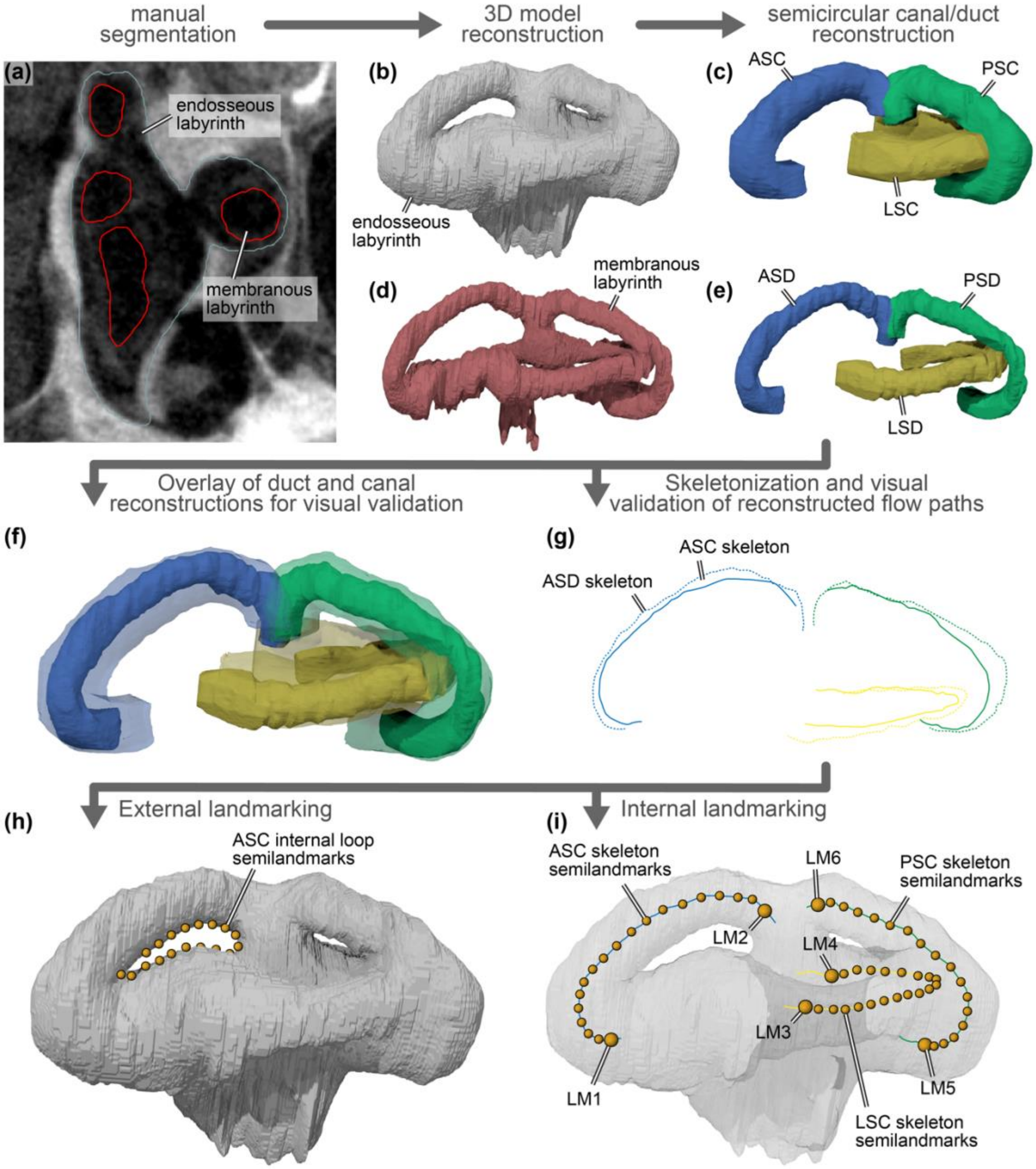

Supplementary Figure 1. Reconstruction and landmarking concept exemplified by *Cuora amboinensis* (SMNS 4867-2). **A**, coronal cross-section of PTA-stained CT scan showing outlines of the left membranous and endosseous labyrinth. **B**, 3D rendering of endosseous labyrinth. **C**, isolated semicircular canals of B. **D**, 3D rendering of membranous labyrinth. **E**, isolated semicircular ducts of D. **F**, isolated semicircular canals and ducts laid over one another for visual comparison. **G**, semicircular canal (solid line) and duct (dashed line) midline skeletons laid over one another for visual comparison. Note that skeleton of membranous ducts represents the actual endolymph flowpath, and that the skeleton of the endosseous canals represents the reconstructed flowpath based on endosseous morphology generally available for extant dry specimens and fossils. **H**, landmarks placed on external surface of endosseous labyrinths. **I**, internal landmarks placed along midline skeletons of semicircular canal midline skeletons, which are reconstructions of the endolymph flowpath. Abbreviations: ASC, anterior semicircular canal; ASD, anterior semicircular duct; LSC, lateral semicircular canal; LSD, lateral semicircular duct; PSC, posterior semicircular canal; PSD, posterior semicircular duct.

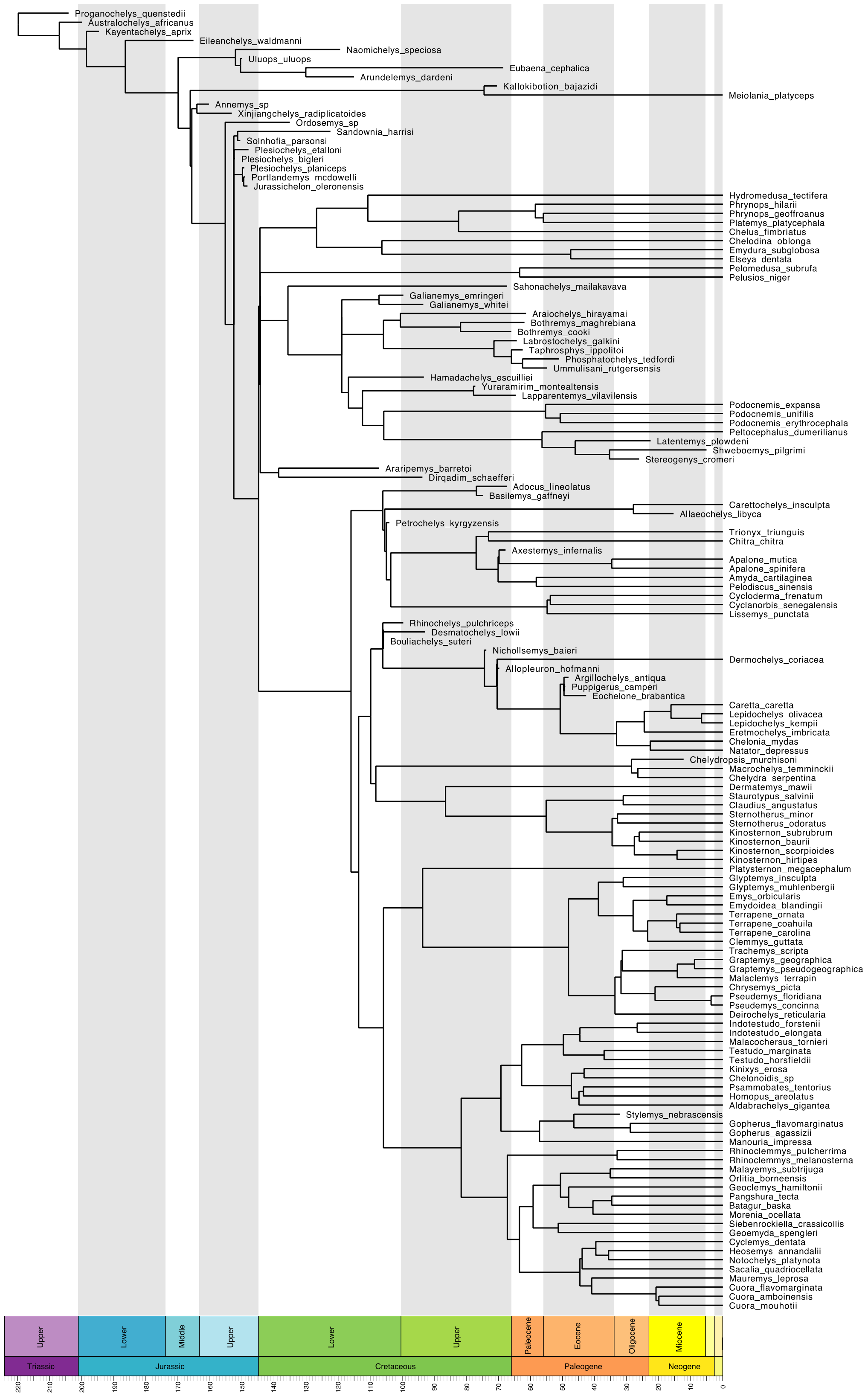

Supplementary Figure 2. Calibrated supertree resulting from cal3-calubration method, which was chosen for main analyses. Note that the tree is deposited as Supplementary Dataset 7.

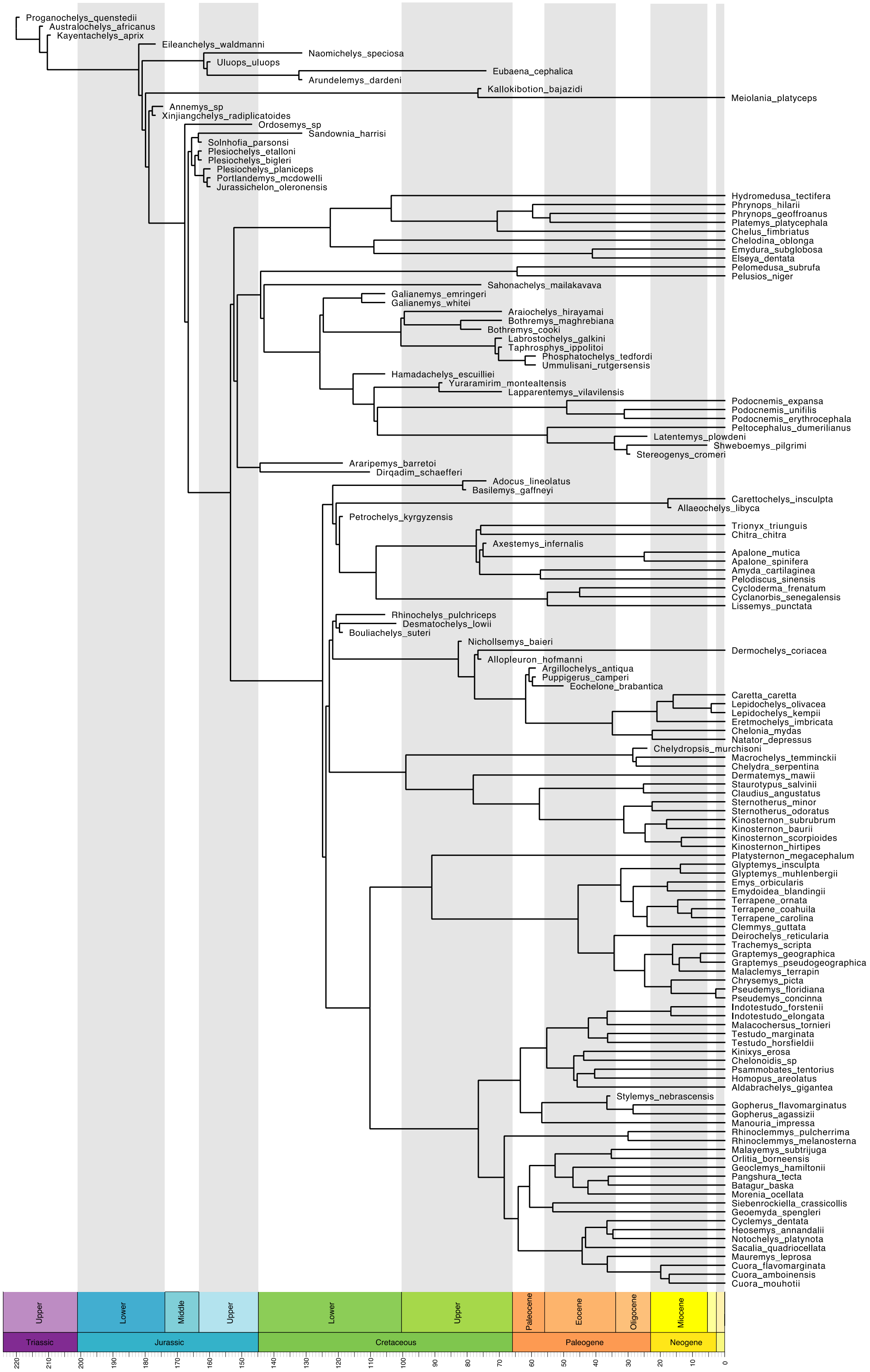

Supplementary Figure 3. Calibrated supertree resulting from mbl-calibration method. This tree was used for sensitivity analyses presented in this supplement. Note that the tree is deposited as Supplementary Dataset 8.

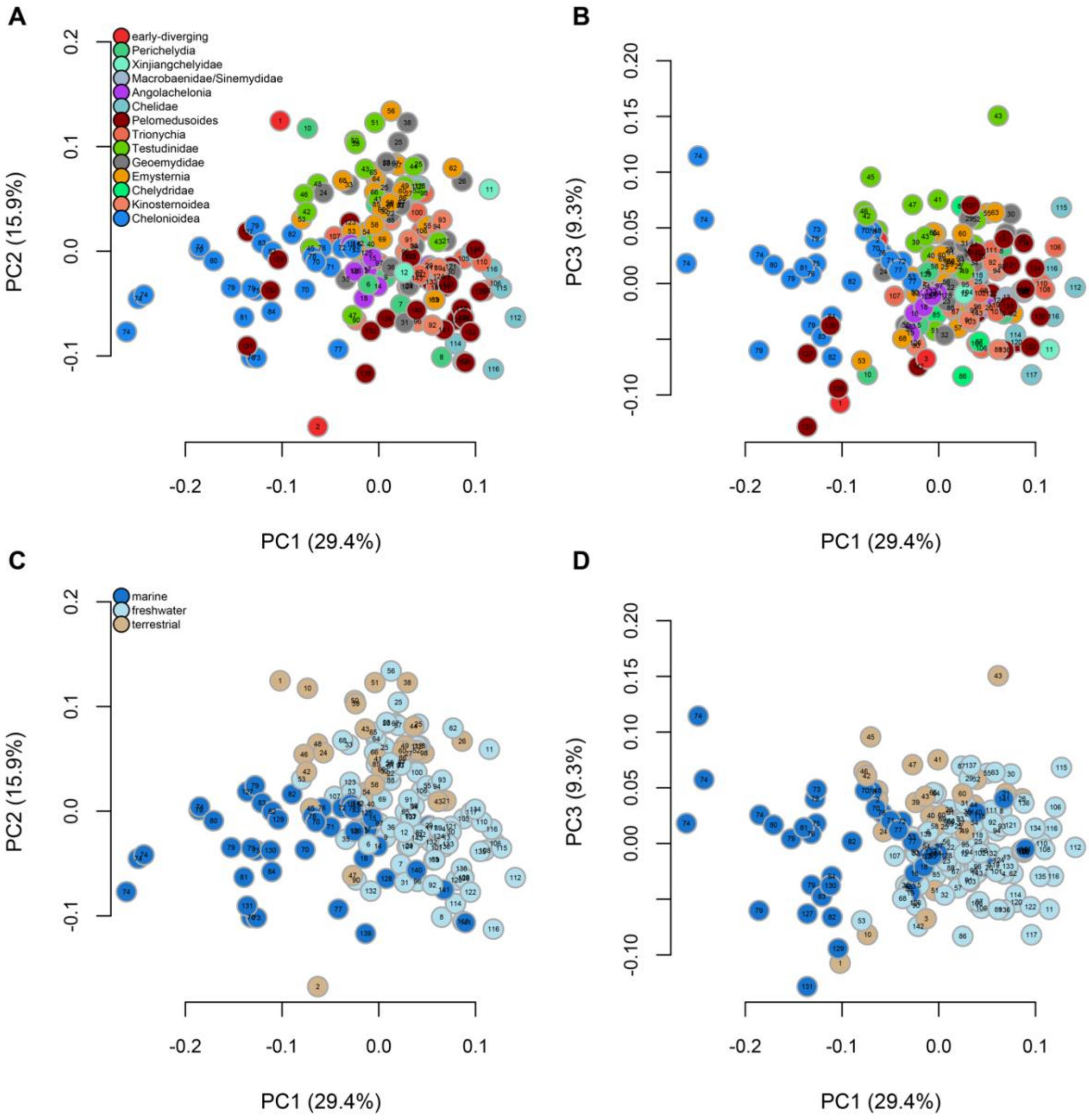

Supplementary Figure 4. Morphospace of turtle labyrinth shape from PCA ordination (N=168). **(a)** PC1 vs. PC2, colour coded by taxonomy. **(b)** PC1 vs. PC3, color coded by taxonomy. **(c)** PC1 vs. PC2, colour coded by ecology. **(d)** PC1 vs. PC3, color coded by ecology. Proportions of total shape variance explained by PC axes is given in brackets. Source data are provided with this paper. Species key: 1 - *Proganochelys quenstedtii*; 2 - *Australochelys africanus*; 3 - *Kayentachelys aprix*; 4 - *Eileanchelys waldmani*; 5 - *Naomichelys speciosa*; 6 - *Uluops uluops*; 7 - *Eubaena cephalica*; 8 - *Arundelemys dardeni*; 9 - *Kallokibotion bajazidi*; 10 - *Meiolania platyceps*; 11 - *Annemys* sp; 12 - *Xinjiangchelys radiplicatoides*; 13 - *Ordosemys* sp; 14 - *Sandownia harrisi*; 15 - *Solnhofia parsonsi*; 16 - *Plesiochelys etalloni*; 17 - *Plesiochelys bigleri*; 18 - *Plesiochelys planiceps*; 19 - *Portlandemys mcdowelli*; 20 - *Jurassichelon oleronensis*; 21 - *Rhinoclemmys pulcherrima*; 22 - *Rhinoclemmys melanosterna*; 23 - *Mauremys leprosa*; 24 - *Cuora flavomarginata*; 25 - *Cuora amboinensis*; 26 - *Cuora mouhotii*; 27 - *Cyclemys dentata*; 28 - *Heosemys annandalii*; 29 - *Notochelys platynota*; 30 - *Sacalia quadriocellata*; 31 - *Malayemys subtrijuga*; 32 - *Orlitia borneensis*; 33 - *Geoclemys hamiltonii*; 34 - *Pangshura tecta*; 35 - *Batagur baska*; 36 - *Morenia ocellata*; 37 - *Siebenrockiella crassicollis*; 38 - *Geoemyda spengleri*; 39 - *Indotestudo forstenii*; 40 - *Indotestudo elongata*; 41 - *Malacochersus tornieri*; 42 - *Testudo marginata*; 43 - *Testudo horsfieldii*; 44 - *Kinixys erosa*; 45 - *Chelonoidis* sp; 46 - *Psammobates tentorius*; 47 - *Homopus areolatus*; 48 - *Aldabrachelys gigantea*; 49 - *Stylemys nebrascensis*; 50 - *Gopherus flavomarginatus*; 51 - *Gopherus agassizii*; 52 - *Manouria impressa*; 53 - *Platysternon*

megacephalum; 54 - *Glyptemys insculpta*; 55 - *Glyptemys muhlenbergii*; 56 - *Emys orbicularis*; 57 - *Emydoidea blandingii*; 58 - *Terrapene ornata*; 59 - *Terrapene coahuila*; 60 - *Terrapene carolina*; 61 - *Clemmys guttata*; 62 - *Deirochelys reticularia*; 63 - *Trachemys scripta*; 64 - *Graptemys geographica*; 65 - *Graptemys pseudogeographica*; 66 - *Malaclemys terrapin*; 67 - *Chrysemys picta*; 68 - *Pseudemys floridana*; 69 - *Pseudemys concinna*; 70 - *Rhinochelys pulchriceps*; 71 - *Desmatochelys lowii*; 72 - *Bouliachelys suteri*; 73 - *Nichollsemys baieri*; 74 - *Dermochelys coriacea*; 75 - *Allopleuron hofmanni*; 76 - *Argillochelys antiqua*; 77 - *Puppigerus camperi*; 78 - *Eochelone brabantica*; 79 - *Caretta caretta*; 80 - *Lepidochelys olivacea*; 81 - *Lepidochelys kempii*; 82 - *Eretmochelys imbricata*; 83 - *Chelonia mydas*; 84 - *Natator depressus*; 85 - *Chelydropsis murchisoni*; 86 - *Macrochelys temminckii*; 87 - *Chelydra serpentina*; 88 - *Dermatemys mawii*; 89 - *Staurotypus salvinii*; 90 - *Claudius angustatus*; 91 - *Sternotherus minor*; 92 - *Sternotherus odoratus*; 93 - *Kinosternon subrubrum*; 94 - *Kinosternon baurii*; 95 - *Kinosternon scorpioides*; 96 - *Kinosternon hirtipes*; 97 - *Adocus lineolatus*; 98 - *Basilemys gaffneyi*; 99 - *Carettochelys insculpta*; 100 - *Allaeochelys libyca*; 101 - *Petrochelys kyrgyzensis*; 102 - *Trionyx triunguis*; 103 - *Chitra chitra*; 104 - *Axestemys infernalis*; 105 - *Apalone mutica*; 106 - *Apalone spinifera*; 107 - *Amyda cartilaginea*; 108 - *Pelodiscus sinensis*; 109 - *Cycloderma frenatum*; 110 - *Cyclanorbis senegalensis*; 111 - *Lissemys punctata*; 112 - *Hydromedusa tectifera*; 113 - *Phrynops hilarii*; 114 - *Phrynops geoffroanus*; 115 - *Platemys platycephala*; 116 - *Chelus fimbriatus*; 117 - *Chelodina oblonga*; 118 - *Emydura subglobosa*; 119 - *Elseya dentata*; 120 - *Pelomedusa subrufa*; 121 - *Pelusios niger*; 122 - *Sahonachelys mailakavava*; 123 - *Galianemys emringeri*; 124 - *Galianemys whitei*; 125 - *Araiochelys hirayamai*; 126 - *Bothremys maghrebiana*; 127 - *Bothremys cooki*; 128 - *Labrostochelys galkini*; 129 - *Taphrosphys ippolitoi*; 130 - *Phosphatochelys tedfordi*; 131 - *Ummulisani rutgersensis*; 132 - *Hamadachelys escuilliei*; 133 - *Yuraramirim montealtensis*; 134 - *Lapparentemys vilavilensis*; 135 - *Podocnemis expansa*; 136 - *Podocnemis unifilis*; 137 - *Podocnemis erythrocephala*; 138 - *Peltocephalus dumerilianus*; 139 - *Latentemys plowdeni*; 140 - *Shweboemys pilgrimi*; 141 - *Stereogenys cromeri*; 142 - *Araripemys barretoi*; 143 - *Dirqadim schaefferi*

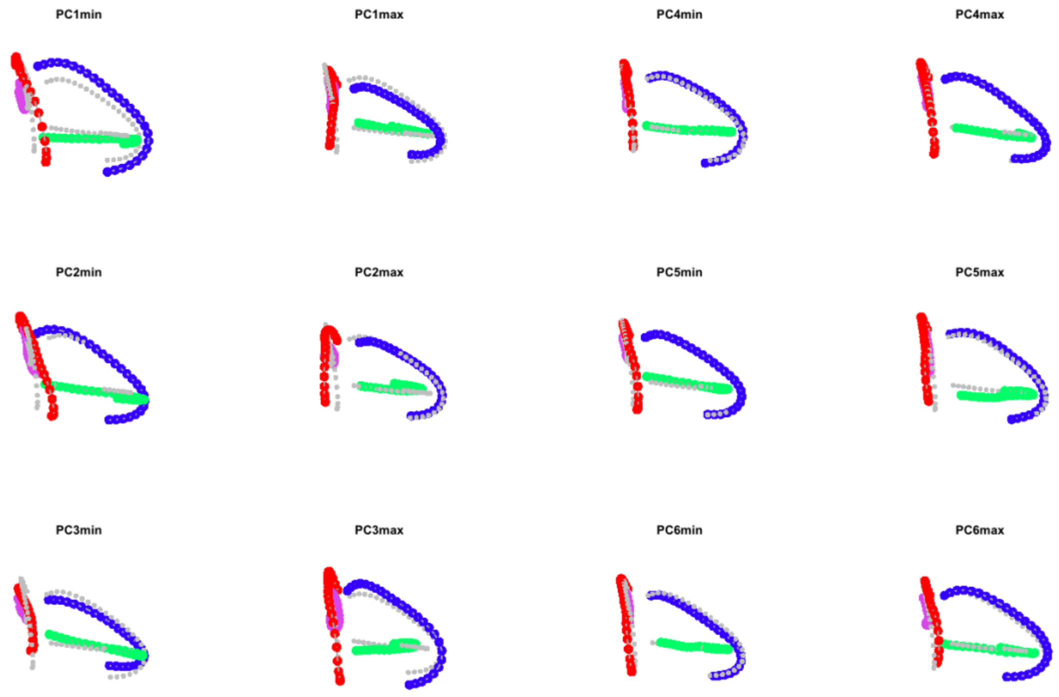

Supplementary Figure 5. Maximum shape deformations of the landmark data in anterior view (i.e., looking from anterior onto loop of PSC) along PC1–6 (colored) against mean shape (grey).

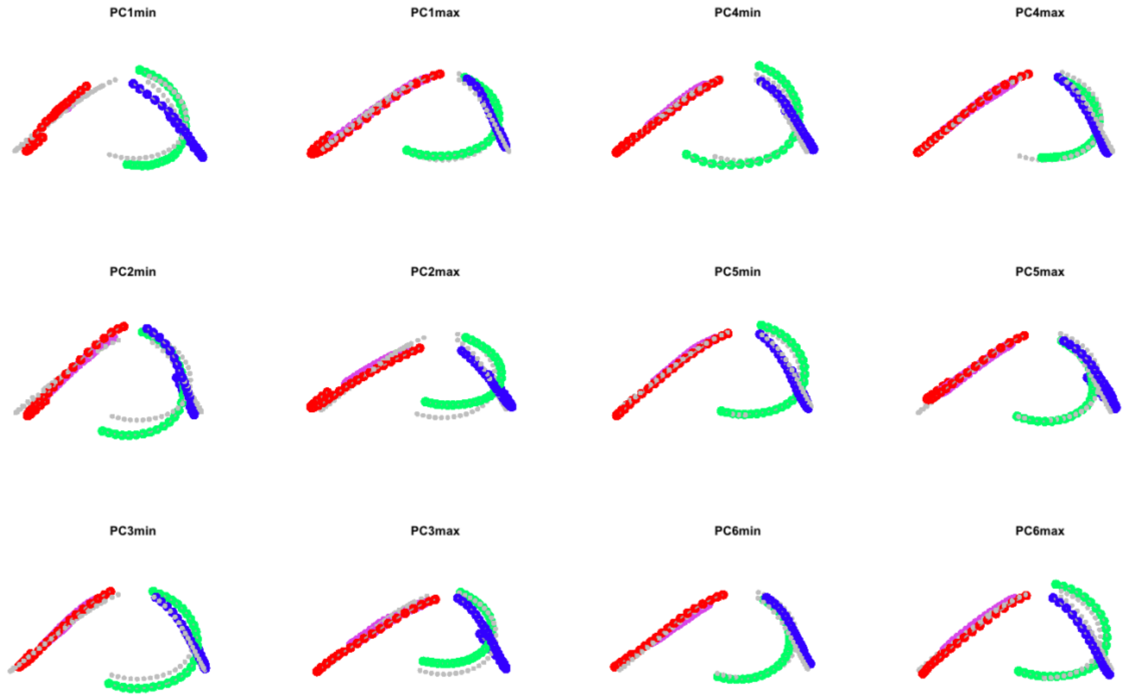

Supplementary Figure 6. Maximum shape deformations of the landmark data in dorsal view along PC1–6 (colored) against mean shape (grey).

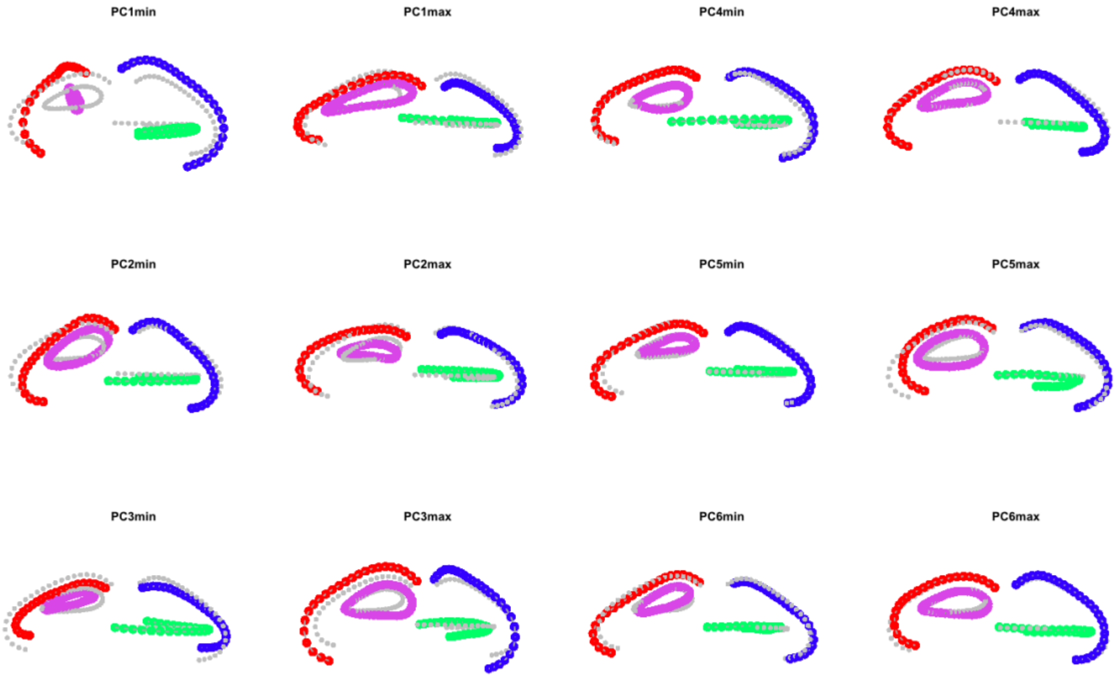

Supplementary Figure 7. Maximum shape deformations of the landmark data in lateral view along PC1–6 (colored) against mean shape (grey).

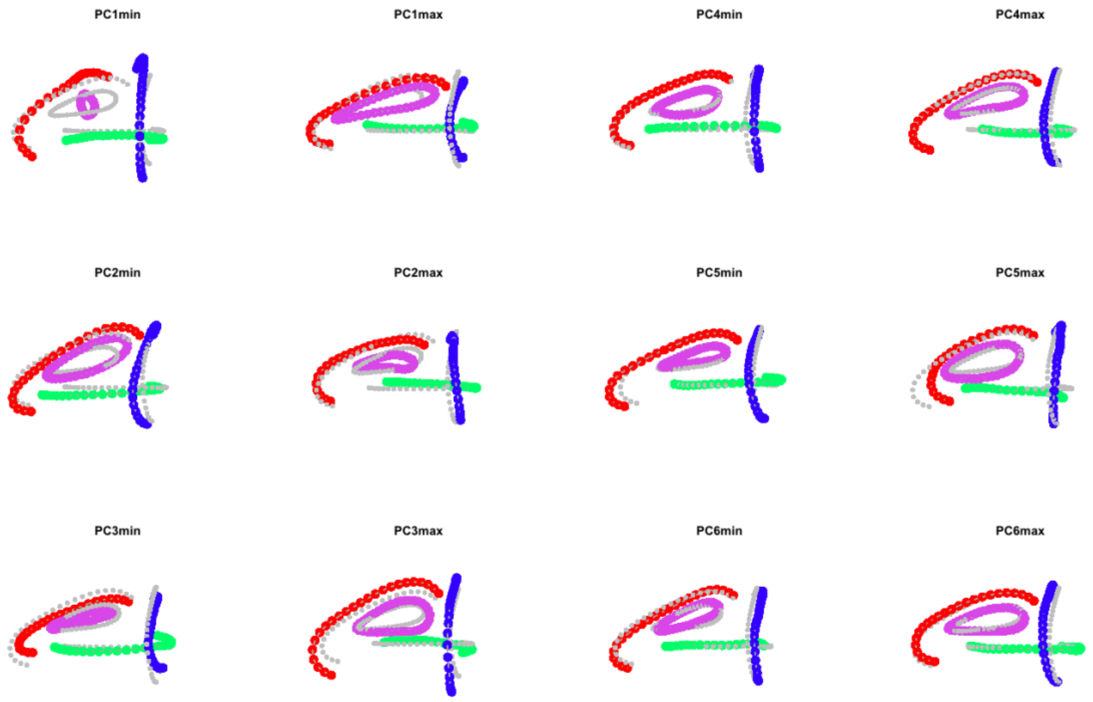

Supplementary Figure 8. Maximum shape deformations of the landmark data in anterior view (i.e., looking from posterior onto loop of ASC) along PC1–6 (colored) against mean shape (grey).

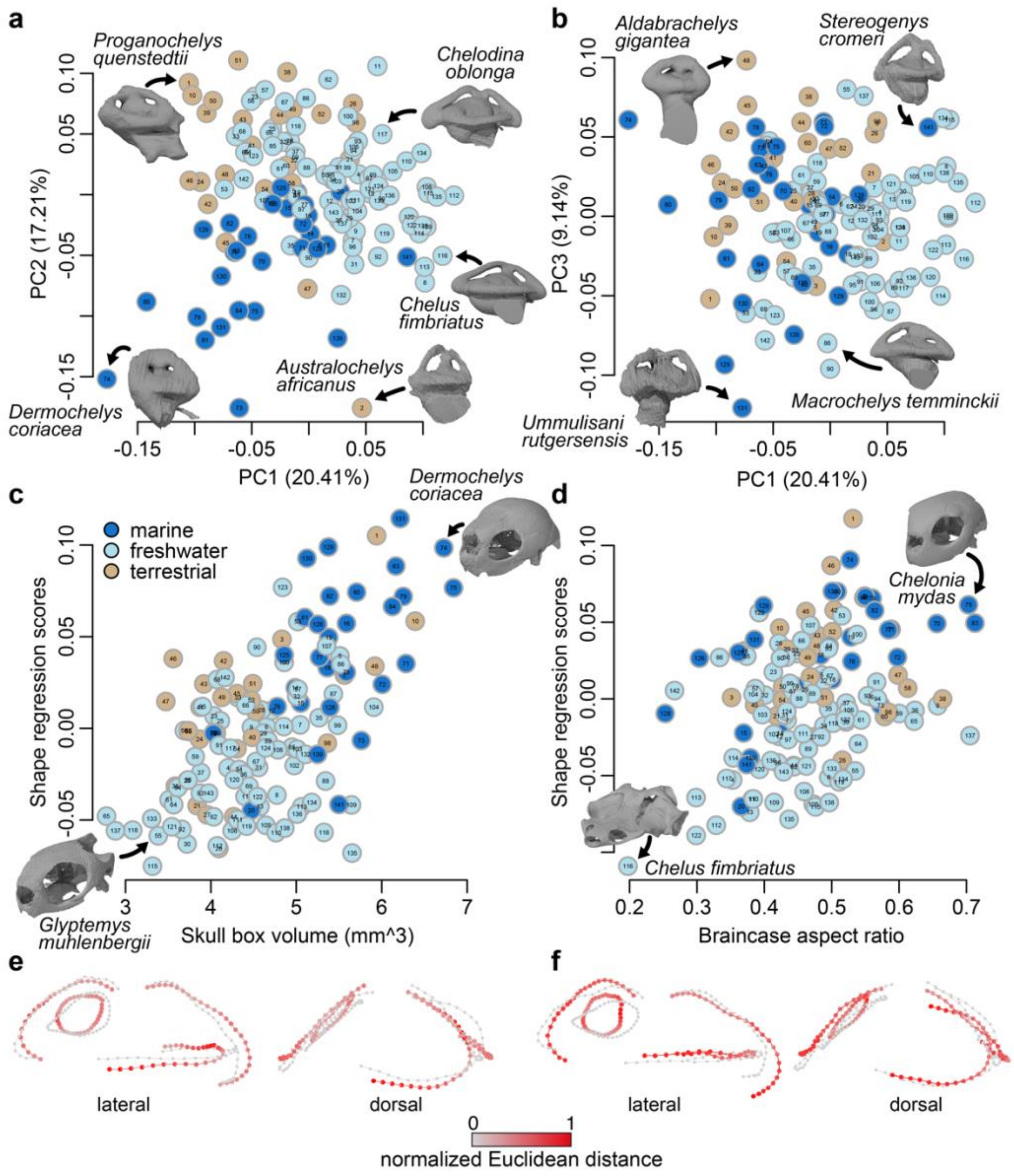

Supplementary Figure 9. Morphospaces of size-corrected labyrinth shape (N=138) and allometric and braincase aspect ratio effects on labyrinth shape. **(a)** PC1 vs. PC2 of labyrinth shape corrected for skull size allometry and braincase aspect ratio **(b)** PC1 vs. PC3 of labyrinth shape corrected for skull size allometry and braincase aspect ratio. Proportions of total shape variance explained by PC axes is given in brackets. **(c)** Plots of skull box volume regression scores against skull box volume, using the formula shape ~ skull box volume + braincase aspect ratio'. Data points are colour coded by ecology. **(d)** Plots of braincase aspect ratio regression scores against skull box volume **(e)** Deformation plots at large (red) and small (grey) skull sizes **(f)** Deformation plots at large (red) and small (grey) brain aspect ratios. Source data are provided with this paper. Species key: 1 - *Proganochelys quenstedtii*; 2 - *Australochelys africanus*; 3 - *Kayentachelys aprix*; 4 - *Eileanchelys waldmani*; 5 - *Naomichelys speciosa*; 6 - *Uluops uluops*; 7 - *Eubaena cephalica*; 8 - *Arundelemys dardeni*; 9 - *Kallokibotion bajazidi*; 10 - *Meiolania platyceps*; 11 - *Annemys* sp; 12 - *Xinjiangchelys radiplicatoides*; 13 - *Ordosemys* sp; 14 - *Sandownia harrisi*; 15 - *Solnhofia parsonsi*; 16 - *Plesiochelys etalloni*; 17 - *Plesiochelys bigleri*; 18 - *Plesiochelys planiceps*; 19 - *Portlandemys mcdowelli*; 20 - *Jurassichelon oleronensis*; 21 - *Rhinoclemmys pulcherrima*; 22 - *Rhinoclemmys melanosterna*; 23 - *Mauremys leprosa*; 24 - *Cuora flavomarginata*; 25 - *Cuora amboinensis*; 26 - *Cuora mouhotii*; 27 - *Cyclemys dentata*; 28 - *Heosemys annandalii*; 29 - *Notochelys platynota*; 30 - *Sacalia quadriocellata*; 31 - *Malayemys subtrijuga*; 32 - *Orlitia borneensis*; 33 - *Geoclemys hamiltonii*; 34 - *Pangshura tecta*; 35 - *Batagur baska*; 36 - *Morenia ocellata*; 37 - *Siebenrockiella crassicollis*; 38 - *Geoemyda spengleri*; 39 - *Indotestudo forstenii*; 40 - *Indotestudo elongata*; 41 - *Malacochersus tornieri*; 42 - *Testudo marginata*; 43 - *Testudo horsfieldii*; 44 - *Kinixys erosa*; 45 - *Chelonoidis* sp; 46 - *Psammobates tentorius*; 47 - *Homopus areolatus*; 48 - *Aldabrachelys gigantea*; 49 - *Stylemys*

nebrascensis; 50 - *Gopherus flavomarginatus*; 51 - *Gopherus agassizii*; 52 - *Manouria impressa*; 53 - *Platysternon megacephalum*; 54 - *Glyptemys insculpta*; 55 - *Glyptemys muhlenbergii*; 56 - *Emys orbicularis*; 57 - *Emydoidea blandingii*; 58 - *Terrapene ornata*; 59 - *Terrapene coahuila*; 60 - *Terrapene carolina*; 61 - *Clemmys guttata*; 62 - *Deirochelys reticularia*; 63 - *Trachemys scripta*; 64 - *Graptemys geographica*; 65 - *Graptemys pseudogeographica*; 66 - *Malaclemys terrapin*; 67 - *Chrysemys picta*; 68 - *Pseudemys floridana*; 69 - *Pseudemys concinna*; 70 - *Rhinochelys pulchriceps*; 71 - *Desmatochelys lowii*; 72 - *Bouliachelys suteri*; 73 - *Nichollsemys baieri*; 74 - *Dermochelys coriacea*; 75 - *Allopleuron hofmanni*; 76 - *Argillochelys antiqua*; 77 - *Puppigerus camperi*; 78 - *Eochelone brabantica*; 79 - *Caretta caretta*; 80 - *Lepidochelys olivacea*; 81 - *Lepidochelys kempii*; 82 - *Eretmochelys imbricata*; 83 - *Chelonia mydas*; 84 - *Natator depressus*; 85 - *Chelydropsis murchisoni*; 86 - *Macrochelys temminckii*; 87 - *Chelydra serpentina*; 88 - *Dermatemys mawii*; 89 - *Staurotypus salvinii*; 90 - *Claudius angustatus*; 91 - *Sternotherus minor*; 92 - *Sternotherus odoratus*; 93 - *Kinosternon subrubrum*; 94 - *Kinosternon baurii*; 95 - *Kinosternon scorpioides*; 96 - *Kinosternon hirtipes*; 97 - *Adocus lineolatus*; 98 - *Basilemys gaffneyi*; 99 - *Carettochelys insculpta*; 100 - *Allaeochelys libyca*; 101 - *Petrochelys kyrgyzensis*; 102 - *Trionyx triunguis*; 103 - *Chitra chitra*; 104 - *Axestemys infernalis*; 105 - *Apalone mutica*; 106 - *Apalone spinifera*; 107 - *Amyda cartilaginea*; 108 - *Pelodiscus sinensis*; 109 - *Cycloderma frenatum*; 110 - *Cyclanorbis senegalensis*; 111 - *Lissemys punctata*; 112 - *Hydromedusa tectifera*; 113 - *Phrynops hilarii*; 114 - *Phrynops geoffroanus*; 115 - *Platemys platycephala*; 116 - *Chelus fimbriatus*; 117 - *Chelodina oblonga*; 118 - *Emydura subglobosa*; 119 - *Elseya dentata*; 120 - *Pelomedusa subrufa*; 121 - *Pelusios niger*; 122 - *Sahonachelys mailakavava*; 123 - *Galianemys emringeri*; 124 - *Galianemys whitei*; 125 - *Araiochelys hirayamai*; 126 - *Bothremys maghrebiana*; 127 - *Bothremys cooki*; 128 - *Labrostochelys galkini*; 129 - *Taphrosphys ippolitoi*; 130 - *Phosphatochelys tedfordi*; 131 - *Ummulisani rutgersensis*; 132 - *Hamadachelys escuilliei*; 133 - *Yuraramirim montealtensis*; 134 - *Lapparentemys vilavilensis*; 135 - *Podocnemis expansa*; 136 - *Podocnemis unifilis*; 137 - *Podocnemis erythrocephala*; 138 - *Peltocephalus dumerilianus*; 139 - *Latentemys plowdeni*; 140 - *Shweboemys pilgrimi*; 141 - *Stereogenys cromeri*; 142 - *Araripemys barretoi*; 143 - *Dirqadim schaefferi*

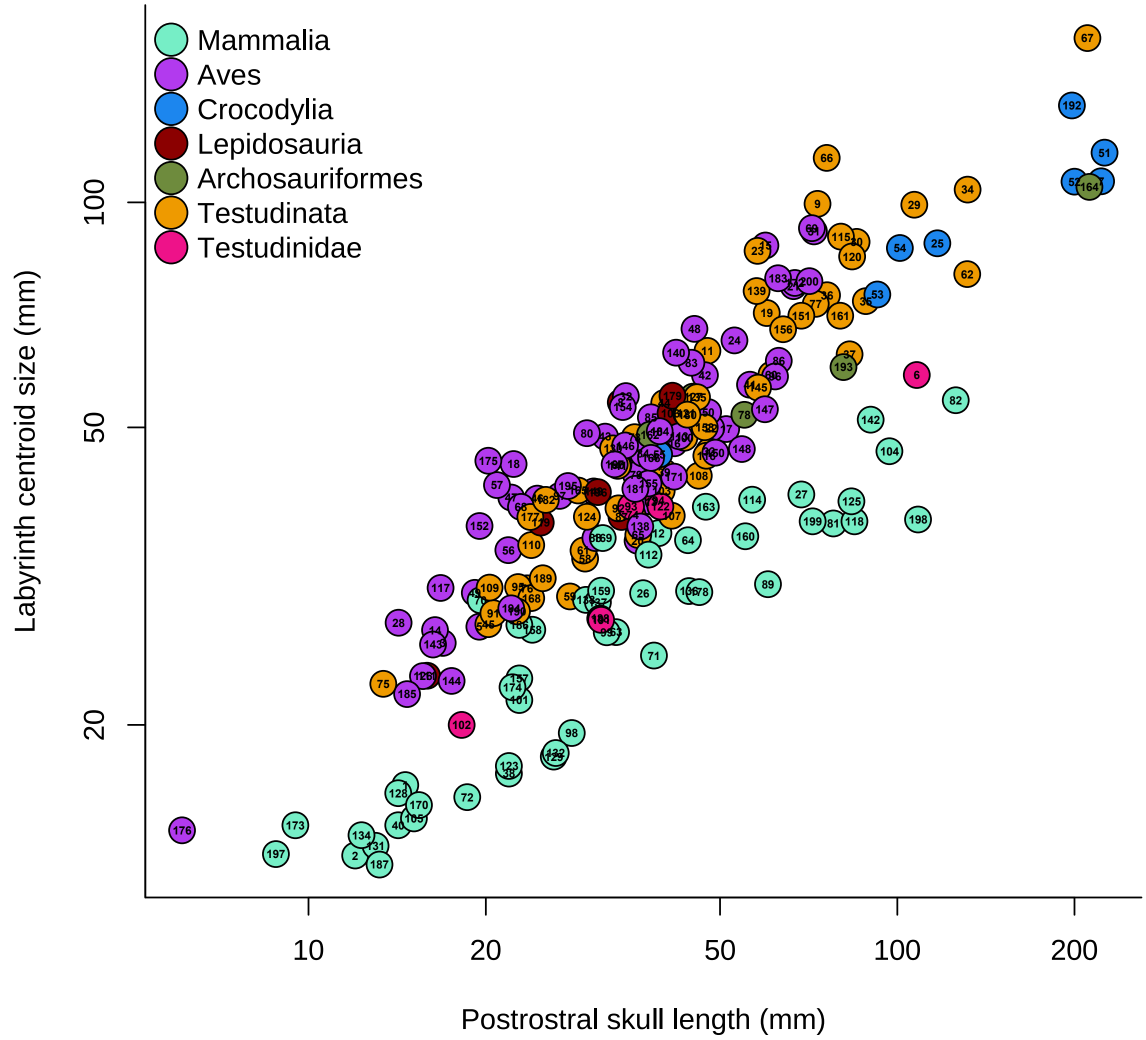

Supplementary Figure 10. Relative labyrinth sizes in tetrapods. Labyrinth centroid size data plotted against postrostral skull lengths. Source data are provided with this paper. Species key: 1 - *Acomys cahirinus*; 2 - *Acrobates pygmaeus*; 3 - *Actitis hypoleucos*; 4 - *Alca torda*; 5 - *Alcedo atthis*; 6 - *Aldabrachelys gigantea*; 7 - *Alligator mississipiensis*; 8 - *Amblyrhynchus cristatus*; 9 - *Amyda cartilaginea*; 10 - *Annemys* sp.; 11 - *Apalone spinifera*; 12 - *Aplodontia rufa*; 13 - *Apteryx hastii*; 14 - *Apus apus*; 15 - *Aquila chrysaetos*; 16 - *Ara macao*; 17 - *Ardea cineria*; 18 - *Athene cunicularia*; 19 - *Australochelys africanus*; 20 - *Aythya fuligula*; 21 - *Balaeniceps rex*; 22 - *Balearica pavonina*; 23 - *Batagur baska*; 24 - *Buteo buteo*; 25 - *Caiman crocodilus*; 26 - *Callimico goeldii*; 27 - *Canis mesomelas*; 28 - *Caprimulgus europaeus*; 29 - *Caretta caretta*; 30 - *Carettochelys insculpta*; 31 - *Casuarius casuarius*; 32 - *Cathartes* sp.; 33 - *Chelodina oblonga*; 34 - *Chelonia mydas*; 35 - *Chelus fimbriatus*; 36 - *Chelydra serpentina*; 37 - *Chitra chitra*; 38 - *Chlamydophorus truncatus*; 39 - *Chrysemys picta*; 40 - *Chrysochloris asiatica*; 41 - *Ciconia ciconia*; 42 - *Ciconia nigra*; 43 - *Circus cyaneus*; 44 - *Claudius angustatus*; 45 - *Clemmys guttata*; 46 - *Columba livia*; 47 - *Coracias garrulous*; 48 - *Corvus corax*; 49 - *Coturnix coturnix*; 50 - *Creagus furcatus*; 51 - *Crocodylus acutus*; 52 - *Crocodylus intermedius*; 53 - *Crocodylus johnstoni*; 54 - *Crocodylus moreletii*; 55 - *Crocodylus porosus*; 56 - *Crypturellus tataupa*; 57 - *Cuculus canorus*; 58 - *Cuora amboinensis*; 59 - *Cuora flavomarginata*; 60 - *Cyclanorbis senegalensis*; 61 - *Cyclemys dentata*; 62 - *Cycloderma frenatum*; 63 - *Cyclopes didactylus*; 64 - *Cynocephalus volans*; 65 - *Deirochelys reticularia*; 66 - *Dermatemys mawii*; 67 - *Dermochelys coriacea*; 68 - *Dicrurus paradiseus*; 69 - *Diomedea exulans*; 70 - *Jaculus jaculus*; 71 - *Dobsonia viridis*; 72 - *Dromiciops australis*; 73 - *Elseya dentata*; 74 - *Emydoidea blandingii*; 75 - *Emydura subglobosa*; 76 - *Emys orbicularis*; 77 - *Eretmochelys*

imbricata; 78 - *Euparkeria capensis*; 79 - *Falco subbuteo*; 80 - *Falco tinnunculus*; 81 - *Felis catus sylvestris*; 82 - *Puma concolor*; 83 - *Fregata magnificens*; 84 - *Fulmaris glacialis*; 85 - *Gallus gallus*; 86 - *Gavia immer*; 87 - *Gecko verticillatus*; 88 - *Gelochelidon nilotica*; 89 - *Genetta genetta*; 90 - *Geoclemys hamiltonii*; 91 - *Geoemyda spengleri*; 92 - *Glyptemys insculpta*; 93 - *Gopherus agassizii*; 94 - *Gopherus flavomarginatus*; 95 - *Graptemys geographica*; 96 - *Grus grus*; 97 - *Haematopus ostralegus*; 98 - *Heliophobius argenteocinereus*; 99 - *Hemiechinus auritus*; 100 - *Heosemys annandalii*; 101 - *Heteromys desmarestianus*; 102 - *Homopus areolatus*; 103 - *Hydromedusa tectifera*; 104 - *Hystrix leucura*; 105 - *Idiurus zenkeri*; 106 - *Iguana iguana*; 107 - *Indotestudo elongata*; 108 - *Kayentachelys aprix*; 109 - *Kinosternon baurii*; 110 - *Kinosternon subrubrum*; 111 - *Lacerta viridis*; 112 - *Laonastes aenigmamus*; 113 - *Larus argentatus*; 114 - *Lemur macaco*; 115 - *Lepidochelys olivacea*; 116 - *Lissemys punctata*; 117 - *Luscinia megarhynchus*; 118 - *Lutra lutra*; 119 - *Lyriocephalus scutatus*; 120 - *Macrochelys temminckii*; 121 - *Malayemys subtrijuga*; 122 - *Manouria impressa*; 123 - *Marmosa murina*; 124 - *Mauremys leprosa*; 125 - *Mellivora ratel*; 126 - *Melopsittacus undulatus*; 127 - *Mesosuchus browni*; 128 - *Microgale drouhardi*; 129 - *Monodelphis domestica*; 130 - *Morenia ocellata*; 131 - *Mus musculus*; 132 - *Mustela nivalis*; 133 - *Myrmecobius fasciatus*; 134 - *Neomys fodiens*; 135 - *Notochelys platynota*; 136 - *Loris tardigradus*; 137 - *Ochotona alpina*; 138 - *Opisthocomus hoazin*; 139 - *Orlitia borneensis*; 140 - *Pandion haliaetus*; 141 - *Pangshura tecta*; 142 - *Papio hamadryas*; 143 - *Passer domesticus*; 144 - *Pelagodroma marina*; 145 - *Peltocephalus dumerilianus*; 146 - *Phaethon lepturus*; 147 - *Phalacrocorax carbo*; 148 - *Phalacrocorax harrisi*; 149 - *Phasianus colchions*; 150 - *Phoenicopterus ruber*; 151 - *Phrynops hilarii*; 152 - *Dendrocopos major*; 153 - *Platysternon megacephalum*; 154 - *Podargus strigoides*; 155 - *Podiceps cristatus*; 156 - *Podocnemis unifilis*; 157 - *Podogymnura truei*; 158 - *Potamogale velox*; 159 - *Potorous tridactylus*; 160 - *Procavia capensis*; 161 - *Proganochelys quenstedtii*; 162 - *Prolacerta broomi*; 163 - *Pronolagus rupestris*; 164 - *Proterosuchus fergusi*; 165 - *Pseudemys floridana*; 166 - *Psittacus erithacus*; 167 - *Rhamphastos discolorus*; 168 - *Rhinoclemmys melanosterna*; 169 - *Rhynchocyon petersi*; 170 - *Rhyncholestes raphanurus*; 171 - *Rhynchotus rufescens*; 172 - *Sagittarius serpentarius*; 173 - *Salpingotulus michaelis*; 174 - *Sciuropterus sagitta*; 175 - *Scolopax rusticola*; 176 - *Selasphorus rufus*; 177 - *Siebenrockiella crassicollis*; 178 - *Solenodon paradoxus*; 179 - *Sphenodon punctatus*; 180 - *Staurotypus salvinii*; 181 - *Steatornis caripensis*; 182 - *Sternotherus minor*; 183 - *Struthio camelus*; 184 - *Tachyeres brachypterus*; 185 - *Taeniopygia guttata*; 186 - *Tamias striatus*; 187 - *Tarsipes rostratus*; 188 - *Carlito syrichta*; 189 - *Terrapene coahuila*; 190 - *Terrapene ornata*; 191 - *Testudo marginata*; 192 - *Tomistoma schlegelii*; 193 - *Triopticus primus*; 194 - *Trogon curucui*; 195 - *Tyto alba*; 196 - *Varanus indicus*; 197 - *Myotis adversus*; 198 - *Vombatus ursinus*; 199 - *Vulpes lagopus*; 200 - *Vultur gryphus*.

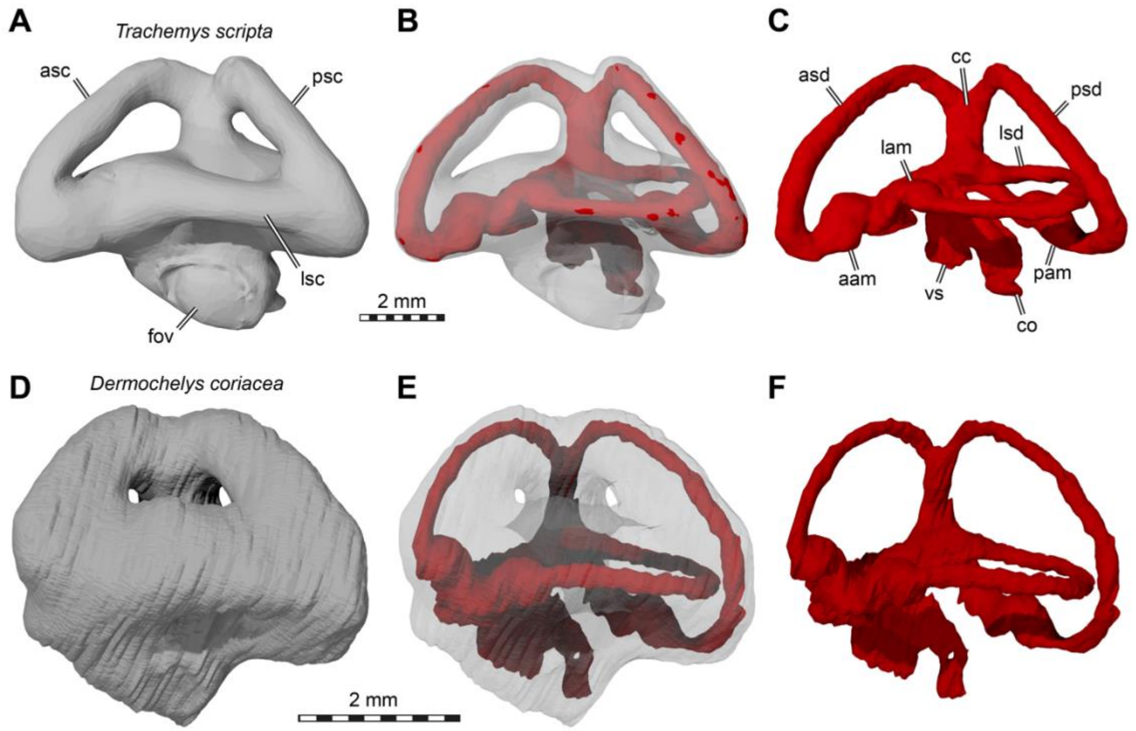

Supplementary Figure 11. Endosseous to membranous labyrinth comparisons. A–C, *Trachemys scripta* (MS-specimen ID376944); D–F, *Dermochelys coriacea* (GPIT 1476). Note how the membranous labyrinth does not show increased internal duct diameters despite the increased canal diameters in *Dermochelys coriacea*, indicating the labyrinth is surrounded by an increased amount of perilymph.

Supplementary Tables

Supplementary Table 1. Neck length data for turtles.

Species	Specimen used for measurement	Cumulative neck length [mm]	Carapace length [mm]	Neck pro-portion [%]	Neck length categorical
Adocus lineolatus	CCM 60-15	179	460	38.91	intermediate
Allopleuron hofmanni	IRSNB 3901	375	900	41.67	intermediate
Amyda cartilaginea	USNM 22522	319.5	319	100.16	extreme
Apalone mutica	USNM 313562	126.7	141	89.86	extreme
Apalone spinifera	YPM R190893	226.4	251	90.2	extreme
Araripemys barretoi	AMNH 24453-4	154.6	230.8	66.98	long
Batagur baska	USNM 226381	229	580	39.48	intermediate
Caretta caretta	AMNH 129869	189.7	585.3	32.41	short
Carettochelys insculpta	CRI 14	193.1	487	39.65	intermediate
Chelodina oblonga	CRI 4632	202.8	263	77.11	extreme
Chelonia mydas	AMNH 5912	237	694	34.15	short
Chelonoidis sp.	CRI 284 (*C. denticulata*)*	208.8	431	48.45	intermediate
Chelus fimbriatus	AMNH 70638	258.4	441	58.59	long
Chitra chitra	inferred from general	NA	NA	NA	extreme
Chrysemys picta	AMNH 75250	65.6	143	45.87	intermediate
Clemmys guttata	USNM 220858	48.9	107	45.7	intermediate
Cuora amboinensis	USNM 241427	61.3	120	51.08	long
Cyclanorbis senegalensis	inferred from general	NA	NA	NA	extreme
Cycloderma frenatum	AMNH 110180	354.2	377	93.95	extreme
Deirochelys reticularia	USNM 80965	166.2	216	76.94	extreme
Dermatemys mawii	SMF 59462	160.7	351	45.78	intermediate
Dermochelys coriacea	approximated from	NA	NA	NA	short
Elseya dentata	QM J59280	64.9	244	26.6	short
Emydoidea blandingii	USNM 220869	135	215	62.79	long
Emydura subglobosa	USNM 231536	81.7	225	36.31	intermediate
Emys orbicularis	MNHN Pal unnumbered	88.6	152	58.29	long
Eochelone brabantica	approximated from various	NA	NA	NA	short
Eretmochelys imbricata	inferred from sister taxon	NA	NA	NA	short
Geoclemys hamiltonii	CRI 487	134.2	262	51.22	long
Gopherus agassizii	USNM 222094	129.3	275	47.02	intermediate
Gopherus flavomarginatus	USNM 51357	166.1	389	42.7	intermediate
Graptemys geographica	FMNH 22080	83	199	41.71	intermediate
Graptemys pseudogeographica	inferred from sister taxon	NA	NA	NA	intermediate
Heosemys annandalii	UCMVZ 241498	114.7	240	47.79	intermediate
Homopus areolatus	OUMNH 9403	37.2	89.1	41.75	intermediate
Hydromedusa tectifera	AMNH 133629	102.5	157	65.29	long
Kinixys erosa	USNM 222517 (*K. belliana*)*	118.8	219	54.25	long
Kinosternon baurii	USNM 167527	56.8	99.5	57.09	long
Kinosternon hirtipes	UFH45035_left	63.3	94.15	67.23	long
Kinosternon scorpioides	inferred from sister taxon	NA	NA	NA	long
Kinosternon subrubruum	inferred from sister taxon	NA	NA	NA	long
Lepidochelys kempii	inferred from sister taxon	NA	NA	NA	short
Lepidochelys olivacea	QM J 85545	162.5	476	34.14	short
Lissemys punctata	NHMUK 172.2066	57.9	81.5	71.04	extreme
Macrochelys temminckii	CRI 6880	261	488	53.48	long
Malacochersus tornieri	USNM 72539	56.6	145	39.03	intermediate
Mauremys leprosa	CRI 6148 (*M. reveesii*)*	102.6	170	60.35	long
Natator depressus	QM J14463	231.3	794	29.13	short
Ordosemys sp	IVPP V9534-1	67.5	272	24.82	short
Pelodiscus sinensis	USNM 539335	90.3	109	82.84	extreme
Pelomedusa subrufa	AMNH 131262	69.6	143	48.67	intermediate
Phrynops geoffroanus	AMNH 79048	174.4	372	46.88	intermediate
Platysternon megacephalum	NCSM76497_left	49.71	106	46.9	intermediate
Podocnemis unifilis	USNM 313861	30.5	113	26.99	short
Podocnemis erythrocephala	PCHP 7525	70.3	216	32.54	short
Puppigerus camperi	IRSNB R 0073	110	346	31.79	short
Sacalia quadriocellata	NCSM 13265	64.58	117.95	54.75	long
Sahonachelys mailakavava	UA 10581	13.6	25.5	53	long
Solnhofia parsonsi	JM SCHA70	50	150	33.33	short
Sternotherus minor	USNM 167534	59.1	90	65.67	long
Sternotherus odoratus	OUMNH 2017070002	61.3	93.6	65.49	long
Terrapene carolina	OUMNH 8795	60.84	115.1	52.86	long
Testudo horsfieldii	OUMNH 10344	73.1	160.2	45.63	intermediate
Testudo marginata	REP 26 MNHN Pal (*T.*	65	153	42.48	intermediate
Trionyx triunguis	AMNH 36599	305.2	291	104.88	extreme
Chelydra serpentina	UF VP1	244	650	37.54	intermediate
*indicates specimens where closely related species were used					

Supplementary Table 2. Results of selected phylogenetic Procrustes distance regressions of labyrinth shape ~ independent variables including fossils, using the alternative tree calibrated with the mbl method. N = 138 for all models. Hypothesis testing used a Procrustes ANOVA, in which statistical significance (*P*-values) is calculated by comparison of sum-of-squared Procrustes distances with sums of squares distributions generated from residual randomization permutation procedure (RRPP[52]), using 1000 permutations. *F*-statistic is the ratio between the sum of squares of the regression and the sum of squares of the error. Effect sizes (*Z*-scores) were computed as standard deviations of *F*-distributions using residual degrees of freedom (re-df). Models presented in same sequence as in Table 1 of main text, which uses a cal3-calibrated tree. Note similarity between results using different trees.

Model	Variable	Effect	*F*-statistic	*Z*-score	*P*-value	R^2	R^2 model	re-df
~ skull box volume *braincase aspect ratio + labyrinth centroid size	skull box volume	allometric	5.009	3.334	0.003	0.032	0.134	133
	braincase aspect ratio	spatial constraint	7.559	4.228	0.001	0.049		
	labyrinth centroid size	allometric	4.924	3.254	0.007	0.032		
	skull box volume: labyrinth centroid size	interaction	3.259	2.327	0.015	0.021		
~ skull box volume *braincase aspect ratio	skull box volume	allometric	4.087	3.041	0.005	0.027	0.096	134
	braincase aspect ratio	spatial constraint	7.465	4.175	0.001	0.05		
	skull box volume: braincase aspect ratio	interaction	2.782	2.008	0.026	0.019		
~ skull box volume * labyrinth centroid size	skull box volume	allometric	5.495	3.472	0.003	0.038	0.078	134
	labyrinth centroid size	allometric	4.385	3.056	0.008	0.03		
	skull box volume: labyrinth centroid size	interaction	1.483	0.968	0.149	0.01		
~ braincase aspect ratio	braincase aspect ratio	spatial constraint	9.132	4.403	0.001	0.063	0.063	136
~ skull height	skull height	allometric	8.749	4.315	0.002	0.06	0.06	136
~ skull box volume	skull box volume	allometric	5.733	3.648	0.002	0.04	0.04	136
~ skull length	skull length	allometric	4.128	3.049	0.003	0.029	0.029	136
~ skull width	skull width	allometric	4.324	3.185	0.002	0.031	0.031	136
~ labyrinth centroid size	labyrinth centroid size	allometric	4.617	3.263	0.004	0.033	0.033	136
~ skull box volume + marine.all	skull box volume	allometric	5.181	3.36	0.004	0.037	0.042	135
	all extant and extinct marine taxa	ecological	0.795	-0.1	0.519	0.005		
~ marine.all	all extant and extinct marine taxa	ecological	1.299	0.77	0.217	0.009	0.009	136
~ marine.extant	extant marine taxa	ecological	0.586	-0.454	0.672	0.004	0.004	136
~ freshwater	Freshwater habitat ecology	ecological	1.339	0.798	0.201	0.01	0.01	136
~ terrestrial	Terrestrial habitat ecology	ecological	1.516	1.009	0.151	0.011	0.011	136
~ incomplete retraction	no ability to retract neck	morphofunctional	1.069	0.376	0.351	0.008	0.008	136
~ full retraction	ability to fully retract neck	morphofunctional	1.069	0.376	0.351	0.008	0.008	136
~ no retraction plane	no preferred plane developed, ancestral anatomy	morphofunctional	0.153	-3.309	1	0.001	0.001	136
~ vertical retraction	cryptodiran neck anatomy	morphofunctional	0.249	-2.312	0.993	0.002	0.002	136
~ horizontal retraction	pleurodiran neck anatomy	morphofunctional	0.368	-1.547	0.948	0.003	0.003	136

Supplementary Table 3. Results of pGLS regressions of labyrinth centroid size ~ independent variables for extant turtles, showing only models with non-negligible AICc values, using the alternative tree calibrated with the mbl method (full table in **Supplementary Data 15**). Models are ordered by AICc rank, showing the best model on top. N = 89 for all models. *λ* **(**lamba) is the phylogenetic signal[53] and was estimated during model fitting. R^2 is the generalized coefficient of determination described by Nagelkerke[54]. Coefficients are estimated using pGLS restricted maximum likelihood. The *t*-statistics are coefficient estimates divided by their standard error. *P*-values are two-sided, and are calculated using the coefficient value and a *t*-distribution with the number of residual degrees of freedom (re-df) of the model. Note similarity between results using different trees when comparing to Table 2 from main text.

**Independent variables**	**Lambda (λ)**	**Variable**	**Coefficient**	***t*-value**	***P*-value**	**re-df**	**AICc**	**AICc weight**	R^2
~ skull box volume + braincase aspect ratio + open water locomotion	0.971	Intercept	0.61	10.000	$5.2*10^{-16}$	85	-267.74	0.224	0.902
		Log_{10}(skull box vol.)	0.21	25.613	$9.9*10^{-42}$				
		braincase aspect ratio	-0.20	-2.981	0.004				
		open water locomotion	0.06	3.243	0.002				
~ skull box volume + forelimbs not webbed	0.902	Intercept	0.50	12.203	$1.8*10^{-20}$	86	-266.67	0.131	0.899
		Log_{10}(skull box vol.)	0.23	30.131	$1.8*10^{-47}$				
		forelimbs not webbed	-0.07	-3.291	0.001				
~ skull box volume	0.993	Intercept	0.49	11.428	$5.2*10^{-19}$	87	-266.55	0.123	0.896
		Log_{10}(skull box vol.)	0.23	30.017	$1.1*10^{-47}$				
~ skull box volume + braincase aspect ratio	0.984	Intercept	0.61	9.374	$8.5*10^{-15}$	86	-266.26	0.107	0.898
		Log_{10}(skull box vol.)	0.22	25.472	$7.9*10^{-42}$				
		braincase aspect ratio	-0.16	-2.362	0.02				
~ skull box volume + open water locomotion	0.985	Intercept	0.48	11.319	$1.0*10^{-18}$	86	-265.07	0.059	0.897
		Log_{10}(skull box vol.)	0.23	30.129	$1.8*10^{-47}$				
		open water locomotion	0.05	2.655	0.009				
~ skull width	0.983	Intercept	0.53	12.208	$1.5*10^{-20}$	87	-265.03	0.058	0.894
		Log_{10}(skull width)	0.63	29.109	$1.3*10^{-46}$				
~ skull box volume + braincase aspect ratio + forelimbs not webbed	0.985	Intercept	0.59	9.413	$7.9*10^{-15}$	85	-264.52	0.045	0.899
		Log_{10}(skull box vol.)	0.22	26.385	$1.0*10^{-42}$				
		braincase aspect ratio	-0.13	-1.942	0.055				
		forelimbs not webbed	-0.06	-2.685	0.009				
~ skull box volume + freshwater habitat ecology	0.872	Intercept	0.44	10.418	$6.5*10^{-17}$	86	-264.43	0.043	0.896
		Log_{10}(skull box vol.)	0.23	30.204	$1.5*10^{-47}$				
		Freshwater habitat ecology	0.04	3.221	0.002				
~ skull box volume + braincase aspect ratio + open water locomotion + forelimbs not webbed	0.935	Intercept	0.60	9.999	$5.9*10^{-16}$	84	-264.10	0.037	0.901
		Log_{10}(skull box vol.)	0.22	26.245	$3.1*10^{-42}$				
		braincase aspect ratio	-0.17	-2.527	0.013				
		open water locomotion	0.05	2.907	0.005				
		forelimbs not webbed	-0.05	-2.254	0.027				
~ skull box volume + terrestrial habitat ecology	0.918	Intercept	0.49	11.885	$7.7*10^{-20}$	86	-263.14	0.022	0.895
		Log_{10}(skull box vol.)	0.23	29.851	$3.7*10^{-47}$				
		terrestrial habitat ecology	-0.04	-2.714	0.008				

Supplementary Table 4. Results of selected phylogenetic Procrustes distance regressions of labyrinth shape ~ independent variables including fossils but excluding all chelonioid sea turtles, using the cal3 tree. N = 123 for all models. Hypothesis testing used a Procrustes ANOVA, in which statistical significance (*P*-values) is calculated by comparison of sum-of-squared Procrustes distances with sums of squares distributions generated from residual randomization permutation procedure (RRPP[52]), using 1000 permutations. *F*-statistic is the ratio between the sum of squares of the regression and the sum of squares of the error. Effect sizes (*Z*-scores) were computed as standard deviations of *F*-distributions using residual degrees of freedom (re-df). Selected models demonstrate absence of independently significant ecological effects.

Model	Variable	Effect	*F*-statistic	*Z*-score	*P*-value	R^2	R^2 model	re-df
~ skull box volume *braincase aspect ratio + labyrinth centroid size	skull box volume	allometric	7.09	3.943	0.001	0.049	0.161	118
	braincase aspect ratio	spatial constraint	7.939	4.448	0.001	0.055		
	labyrinth centroid size	allometric	5.478	3.558	0.001	0.038		
	skull box volume: labyrinth centroid size	interaction	2.804	2.3	0.016	0.019		
~ skull box volume *braincase aspect ratio + labyrinth centroid size + marine.all	skull box volume	allometric	6.69	3.884	0.001	0.046	0.166	117
	braincase aspect ratio	spatial constraint	8.033	4.473	0.001	0.055		
	labyrinth centroid size	allometric	5.613	3.599	0.001	0.039		
	all non-chelonioid marine taxa	ecological	1.14	0.483	0.304	0.008		
	skull box volume: braincase aspect ratio	interaction	2.665	2.203	0.019	0.018		
~ skull box volume *braincase aspect ratio + labyrinth centroid size + freshwater	skull box volume	allometric	6.711	3.828	0.001	0.046	0.162	117
	braincase aspect ratio	spatial constraint	7.953	4.437	0.001	0.055		
	labyrinth centroid size	allometric	5.113	3.412	0.002	0.035		
	Freshwater habitat ecology	ecological	1.012	0.252	0.386	0.007		
	skull box volume: labyrinth centroid size	interaction	2.818	2.331	0.016	0.019		
~ skull box volume *braincase aspect ratio + labyrinth centroid size + terrestrial	skull box volume	allometric	7.56	3.966	0.001	0.052	0.177	117
	braincase aspect ratio	spatial constraint	8.155	4.504	0.001	0.056		
	labyrinth centroid size	allometric	5.565	3.548	0.001	0.038		
	Terrestrial habitat ecology	ecological	1.99	1.607	0.057	0.013		
	skull box volume: labyrinth centroid size	interaction	2.645	2.203	0.018	0.018		
~ braincase aspect ratio	braincase aspect ratio	spatial constraint	11.534	4.669	0.001	0.087	0.087	122
~ skull height	skull height	allometric	12.7	4.852	0.001	0.095	0.095	122
~ skull box volume	skull box volume	allometric	9.025	4.359	0.001	0.069	0.069	122
~ labyrinth centroid size	labyrinth centroid size	allometric	5.272	30351	0.001	0.042	0.042	122
~ marine.all	all extant and extinct	ecological	1.769	1.217	0.114	0.014	0.014	122
~ freshwater	Freshwater habitat	ecological	1.486	1.048	0.149	0.012	0.012	122
~ terrestrial	Terrestrial habitat	ecological	1.612	1.195	0.124	0.013	0.013	122
~ incomplete retraction	no ability to retract neck	morphofunctional	1.448	0.941	0.175	0.012	0.012	122
~ full retraction	ability to fully retract	morphofunctional	1.448	0.941	0.175	0.012	0.012	122
~ no retraction plane	no preferred plane, ancestral anatomy	morphofunctional	0.469	-0.983	0.83	0.004	0.004	122
~ vertical retraction	cryptodiran anatomy	morphofunctional	0.272	-2.073	0.99	0.002	0.002	122
~ horizontal retraction	pleurodiran anatomy	morphofunctional	0.582	-0.815	0.78	0.005	0.005	122

Supplementary Table 5. Results of selected phylogenetic Procrustes distance regressions of labyrinth shape ~ independent variables excluding all marine turtles, using the cal3 tree. N = 109 for all models. Hypothesis testing used a Procrustes ANOVA, in which statistical significance (*P*-values) is calculated by comparison of sum-of-squared Procrustes distances with sums of squares distributions generated from residual randomization permutation procedure (RRPP[52]), using 1000 permutations. *F*-statistic is the ratio between the sum of squares of the regression and the sum of squares of the error. Effect sizes (*Z*-scores) were computed as standard deviations of *F*-distributions using residual degrees of freedom (re-df). Selected models demonstrate absence of independently significant ecological effects.

**Model**	**Variable**	**Effect**	***F*-statistic**	***Z*-score**	***P*-value**	**R^2**	**R^2 model**	**re-df**
~ skull box volume *braincase aspect ratio + labyrinth centroid size	skull box volume	allometric	3.026	2.819	0.002	0.026	0.115	104
	braincase aspect ratio	spatial constraint	4.004	3.342	0.001	0.035		
	labyrinth centroid size	allometric	3.322	2.99	0.001	0.029		
	skull box volume: [illegible]	interaction	2.904	2.358	0.014	0.025		
~ skull box volume *braincase aspect ratio + labyrinth centroid size + freshwater	skull box volume	allometric	2.384	2.293	0.013	0.02	0.109	103
	braincase aspect ratio	spatial constraint	3.984	3.359	0.001	0.034		
	labyrinth centroid size	allometric	2.373	2.248	0.012	0.02		
	Freshwater habitat ecology	ecological	1.497	1.086	0.14	0.013		
	skull box volume: labyrinth centroid size	interaction	2.607	2.181	0.016	0.022		
~ skull box volume *braincase aspect ratio + labyrinth centroid size + terrestrial	skull box volume	allometric	2.384	2.293	0.013	0.02	0.109	103
	braincase aspect ratio	spatial constraint	3.984	3.359	0.001	0.034		
	labyrinth centroid size	allometric	2.373	2.248	0.012	0.02		
	Terrestrial habitat ecology	ecological	1.497	1.086	0.14	0.013		
	skull box volume: labyrinth centroid size	interaction	2.607	2.181	0.016	0.022		
~ braincase aspect ratio	braincase aspect ratio	spatial constraint	3.456	3.127	0.001	0.031	0.031	107
~ skull height	skull height	allometric	2.071	1.811	0.033	0.019	0.095	107
~ skull box volume	skull box volume	allometric	1.658	1.336	0.087	0.015	0.069	107
~ labyrinth centroid size	labyrinth centroid size	allometric	1.848	1.537	0.068	0.017	0.017	107
~ freshwater	Freshwater habitat ecology	ecological	2.446	2.043	0.03	0.022	0.02	107
~ terrestrial	Terrestrial habitat ecology	ecological	2.446	2.043	0.03	0.022	0.022	107
~ incomplete retraction	no ability to retract neck	morpho-functional	1.024	0.39	0.325	0.009	0.009	107
~ full retraction	ability to fully retract neck	morpho-functional	1.024	0.39	0.325	0.012	0.0009	107
~ no retraction plane	no preferred plane developed, ancestral [illegible]	morpho-functional	0.727	-0.334	0.609	0.007	0.007	107
~ vertical retraction	cryptodiran neck anatomy	morpho-functional	0.494	-1.262	0.913	0.005	0.005	107
~ horizontal retraction	pleurodiran neck anatomy	morpho-functional	0.766	-0.341	0.629	0.007	0.007	107

Supplementary Table 6. Results of selected phylogenetic Procrustes distance regressions of labyrinth shape ~ independent variables including fossils, using the cal3 tree and a reduced landmark scheme that excludes the internal ASC loop. N = 138 for all models. Hypothesis testing used a Procrustes ANOVA, in which statistical significance (*P*-values) is calculated by comparison of sum-of-squared Procrustes distances with sums of squares distributions generated from residual randomization permutation procedure (RRPP[52]), using 1000 permutations. *F*-statistic is the ratio between the sum of squares of the regression and the sum of squares of the error. Effect sizes (*Z*-scores) were computed as standard deviations of *F*-distributions using residual degrees of freedom (re-df). Models presented in same sequence as in Table 1 of main text. Note similarity between results.

Model	Variable	Effect	*F*-statistic	*Z*-score	*P*-value	R^2	R^2 model	re-df
~ skull box volume *braincase aspect ratio + labyrinth centroid size	skull box volume	allometric	3.857	3.0	0.006	0.024	0.129	133
	braincase aspect ratio	spatial constraint	7.948	4.457	0.001	0.05		
	labyrinth centroid size	allometric	3.254	2.608	0.009	0.021		
	skull box volume: labyrinth centroid size	interaction	5.398	3.398	0.002	0.034		
~ braincase aspect ratio	braincase aspect ratio	spatial constraint	10.411	4.709	0.001	0.071	0.071	136
~ skull height	skull height	allometric	9.807	4.633	0.001	0.067	0.067	136
~ skull box volume	skull box volume	allometric	7.692	4.271	0.001	0.054	0.054	136
~ skull length	skull length	allometric	7.116	4.16	0.001	0.05	0.05	136
~ skull width	skull width	allometric	5.91	3.823	0.001	0.042	0.042	136
~ labyrinth centroid size	labyrinth centroid size	allometric	0.025	3.786	0.001	0.042	0.042	136
~ marine.all	all extant and extinct marine taxa	ecological	1.07	0.395	0.347	0.008	0.008	136
~ marine.extant	extant marine taxa	ecological	0.675	-0.315	0.615	0.005	0.005	136
~ freshwater	Freshwater habitat ecology	ecological	0.967	0.125	0.456	0.007	0.007	136
~ terrestrial	Terrestrial habitat ecology	ecological	1.345	0.777	0.216	0.01	0.01	136
~ incomplete retraction	no ability to retract neck	morphofunctional	1.682	1.246	0.016	0.012	0.012	136
~ full retraction	ability to fully retract neck	morphofunctional	1.682	1.246	0.106	0.012	0.012	136
~ no retraction plane	no preferred plane developed, ancestral anatomy	morphofunctional	0.271	-1.962	0.98	0.002	0.002	136
~ vertical retraction	cryptodiran neck anatomy	morphofunctional	0.284	-2.059	0.988	0.002	0.002	136
~ horizontal retraction	pleurodiran neck anatomy	morphofunctional	0.48	-1.185	0.88	0.004	0.004	136

Supplementary References

1. Brinkman, D. B. *et al.* “Turtles from the Jurassic Shishigou Formation of the Junggar Basin, People’s Republic of China, with comments on the basicranial region of basal Eucryptodies” in Morphology and Evolution of Turtles, Brinkman, D. B., Holroyd, P. A., Gardner, J. D., Eds. (Springer Science+Business Media, 2013)

2. Gaffney, E. S., Tong, H. & Meylan, P. A. Evolution of the side-necked turtles: the families Bothremydidae, Euraxemydidae, and Araripemydidae. *Bull. Am. Mus. of Nat. Hist.* **300**, 1–700 (2006)

3. Gaffney, E. S. & Jenkins Jr., F. A. The cranial morphology of *Kayentachelys*, an Early Jurassic cryptodire, and the early history of turtles. *Acta Zool.* **91**, 335–368 (2010)

4. Gaffney, E. S., Meylan, A. A., Wood, R. C., Simons, E. & De Almeida Campos, D. Evolution of the side-necked turtles of the family Podocnemididae. *Bull. Am. Mus. of Nat. Hist.* **350**, 1–237 (2011)

5. Evers, S. W. *et al.* Neurovascular anatomy of the protostegid turtle *Rhinochelys pulchriceps* and comparisons of membranous and endosseous labyrinth shape in an extant turtle. *Zool. J. Linn. Soc.* **187**, 800–828 (2019a)

6. Bronzati, M. *et al*. Deep evolutionary diversification of semicircular canals in archosaurs. *Curr. Biol.* **31**(12), 2520–2529 (2021)

7. Pritchard, P. C. H. Piscivory in turtles, and evolution of the long-necked Chelidae. *Symp. Zool. Soc. Long.* **52**, 87–110 (1984)

8. Joyce, W. G. *et al.* A new pelomedusoid turtle, *Sahonachelys mailakavava*, from the Late Cretaceous of Madagascar provides evidence for convergent evolution of specialized suction feeding among pleurodires. *R. Soc. Open Sci.* **8**: 210098 (2021a)

9. Herrel, A., Van Damme, J. & Aerts, P. "Cervical anatomy and function in turtles" in Biology of Turtles, Wyneken, J., Godfrey, M. H., Bels, V., Eds. (CRC Press. 2008), pp. 163-185.

10. Werneburg, I. Neck motion in turtles and its relation to the shape of the temporal skull region. *C. R. Palevol.* **14**, 527–548 (2015)

11. Werneburg I., *et al.* Modeling neck mobility in fossil turtle. *J. Exp. Zool. (Mol. Dev. Evolutionary.)* **324**B: 230–243 (2015)

12. Ferreira, G. S. & Werneburg, I. “Evolution, diversity, and development of the craniocervical system in turtles with special reference to jaw musculature” in Heads, Jaws and Muscles: Evolution, development, anatomical diversity and function, Ziermann, J., Diaz Jr, R. R., Diogo, R., Eds. (Springer. 2019), pp. 171–206 doi: 10.1007/978-3-319-93560-7_8

13. Lautenschlager, S., Ferreira, G. S. & Werneburg, I. Sensory Evolution and Ecology of Early Turtles Revealed by Digital Endocranial Reconstructions. *Front. Ecol. Evol.* **6**, 1–7 (2018)

14. Ferreira, G. S. *et al.* Feeding biomechanics suggests progressive correlation of skull architecture and neck evolution in turtles. *Sci. Rep.* **10**, 5505 (2020)

15. Evers, S. W. & Benson, R. B. J. A new phylogenetic hypothesis of turtles with implications for the timing and number of evolutionary transitions to marine lifestyles in the group. *Palaeontology* **62**(1), 93–134 (2019)

16. Ferreira, G. S., Bronzati, M., Langer, M. C. & Sterli, J. Phylogeny, biogeography and diversification patterns of side-necked turtles (Testudines: Pleurodira). *R. Soc. open sci.* **5**: 171773 (2018)

17. Gaffney, E. S. & Kitching, J. W. The morphology and relationships of *Australochelys*, an Early Jurassic turtle from South Africa. *Am. Mus. Nov.* **3230**, 1–29 (1995)

18. Gaffney, E. S. The comparative osteology of the Triassic turtle *Proganochelys*. *Bull. Am. Mus. of Nat. Hist.* **194**, 1–263 (1990)

19. Joyce, W. G. & Gauthier, J. A. Palaeoecology of Triassic stem turtles sheds new light on turtle origins. *Proc. R. Soc. Long. B* **271**, 1–5 (2004)

20. Joyce, W. G. A review of the fossil record of basal Mesozoic turtles. Bull. *Peabody Mus. Nat. Hist.* **58**(1), 65–113 (2017)

21. Gaffney, E. S. The postcranial morphology of *Meiolania platyceps* and a review of the Meiolaniidae. *Bull. Am. Mus. Nat. Hist.* **229**, 1–165 (1996)

22. Scheyer, T. M. Comparative bone histology of the turtle shell (carapace and plastron): implications for turtle systematics, functional morphology and turtle origins, Dissertation, 1–343 (2007)

23. Sterli, J. A Review of the Fossil Record of Gondwanan Turtles of the Clade Meiolaniformes. *Bull. Peabody Mus. Nat. Hist.* **56**(1), 21–45 (2015)

24. Hutchison, J. H. & Archibald, J. D. Diversity of turtles across the Cretaceous/Tertiary boundary on northeastern Montana. *Palaeogeogr. Palaeoclimatol. Palaeoecol.* **55**, 1–22 (1986)

25. Auffenberg, W. A redefinition of the fossil tortoise genus *Stylemys* Leidy, *J. Paleontol.* **38**(2), 316–324 (1964).

26. Foth, C. *et al.* Comparative analysis of the shape and size of the middle ear cavity of turtles reveals no correlation with habitat ecology. *J. Anat.* **235**, 1078–1097 (2019). https://doi.org/10.1111/joa.13071

27. Pereira, A. G., Sterli, J., Moreira, F. R. R. & Schrago, C. G. Multilocus phylogeny and statistical biogeography clarify the evolutionary history of major lineages of turtles. *Mol. Phylogenet. Evol.* **113**, 59–66 (2017)

28. Evers, S. W., Barrett, P. M. & Benson, R. B. J. Anatomy of *Rhinochelys pulchriceps* (Protostegidae) and marine adaptation during the early evolution of chelonioids. *PeerJ* **7**:e6811 (2019b)

29. Evers, S. W. & Joyce, W. G. A re-description of *Sandownia harrisi* (Testudinata: Sandownidae) from the Aptian of the Isle of Wight based on computed tomography scans. *R. Soc. open sci.* **7**: 191936 (2020)

30. Joyce, W. G., Mäuser, M. & Evers, S. W. Two turtles with soft tissue preservation from the platy limestones of Germany provide evidence for marine flipper adaptations in Late Jurassic thalassochelydians. *PLoS ONE* **16**(6), e0252355 (2021b)

31. Lyson, T. R. & Joyce, W. G. Cranial Anatomy and Phylogenetic Placement of the Enigmatic Turtle *Compsemys victa* Leidy, 1856. *J. Paleontol.* **85**(4), 789-801 (2011)

32. Evers, S. W., Rollot, Y. & Joyce, W. G. Cranial osteology of the Early Cretaceous turtle *Pleurosternon bullockii* (Paracryptodira: Pleurosternidae). *PeerJ* **8**:e9454 (2020)

33. Rollot, Y., Evers, S. W. & Joyce, W. G. A redescription of the Late Jurassic (Tithonian) turtle *Uluops uluops* and a new phylogenetic hypothesis of *Paracryptodira*. *Swiss J. Paleontol.* **140**(23):130 (2021)

34. Sterli, J., Pol, D. & Laurin, M. Incorporating phylogenetic uncertainty on phylogeny-based palaeontological dating and the timing of turtle diversification. *Cladistics* **29**, 233–246 (2013)

35. Joyce, W. G. Phylogenetic relationships of Mesozoic turtles. *Bull. Peabody Mus. Nat. Hist*. **48**(1):3102.

36. Rabi, M., Zhou, C.-F., Wings, O., Ge, S. & Joyce, W. G. A new xinjiangchelyid turtle from the Middle Jurassic of Xinjiang, China and the evolution of the basipterygoid process in Mesozoic turtles. *BMC Evol. Biol.* **13**(203), 1–28 (2013)

37. Brinkman, D. B. *et al*. New exceptionally well-preserved specimens of "*Zangerlia*" *neimongolensis* from Bayan Mandahu, Inner Mongolia, and their taxonomic significance. *C. R. Palevol.* **14**, 577–587 (2015)

38. Mallon, J. C. & Brinkman, D. B. *Basilemys morrinensis*, a new species of nanhsiungchelyid turtle from the Horseshoe Canyon Formation (Upper Cretaceous) of Alberta, Canada. *J. Vertebr. Paleontol.* **38**(2), e1431922 (2018)

39. Joyce, W. G., Rabi, M., Clark, J. M. & Xu, X. A toothed turtle from the Late Jurassic of China and the global biogeographic history of turtles. *BMC Evol. Biol.* **16**, 236 (2016)

40. Brinkman, D. B., Rabi, M. & Zhao, L. Lower Cretaceous fossils from China shed light on the ancestral body plan of crown soft-shell turtles (Trionychidae, Cryptodira). *Sci. Rep.* **7**: 6719 (2017)

41. Vitek, N. S. Giant fossil soft-shelled turtles of North America. *Palaeontol. Electron.* **15**(1), 1–43 (2012)

42. Williams, E. *Testudo cubensis* and the evolution of western hemisphere tortoises. *Bull. Am. Mus. Nat. Hist.* **95**(1), 1–36 (1950)

43. Vlachos, E. A Review of the Fossil Record of North American Turtles of the Clade Pan-Testudinoidea. *Bull. Peabody Mus. Nat. Hi.s* **59**(1):3–94 (2018)

44. Vlachos, E. & Rabi, M. Total evidence analysis and body size evolution of extant and extinct tortoises (Testudines: Cryptodira: Pan-Testudinidae). *Cladistics* **34**, 652–683 (2018)

45. Raselli, I. Comparative cranial morphology of the Late Cretaceous protostegid sea turtle *Desmatochelys lowii*. *PeerJ* **6**:e5964 (2018)

46. Bapst, D. W. A stochastic rate-calibrated method for time-scaling phylogenies of fossil taxa. *Methods Ecol. Evol.* **4**, 724–733 (2013).

47. Laurin, M. The evolution of body size, Cope's Rule and the origin of amniotes. *Syst. Biol.* **53**(4), 594–622 (2004)

48. Bapst, D. W. paleotree: an R package for paleontological and phylogenetic analyses of evolution. *Methods Ecol. Evol.* **3**(5), 803-807 (2012).

49. Bell, M. A. & Lloyd, G. T. strap: an R package for plotting phylogenies against stratigraphy and assessing their stratigraphic congruence. *Palaeontology* 58(2), 379–389 (2015)

50. Lloyd, G. T. Estimating morphological diversity and tempo with discrete character-taxon matrices: implementation, challenges, progress, and future directions. *Biol. J. Linn. Soc.* **118**, 131–151 (2016)

51. Paradis, E. & Schliep, K. ape 5.0: an environment for modern phylogenetics and evolutionary analyses in R. *Bioinform.* **35**, 526-528 (2019)

52. Collyer, M.L., Sekora, D.J. & Adams D.C. A method for analysis of phenotypic change for phenotypes described by high-dimensional data. *Heredity* **115**, 357-365 (2015)

53. Pagel, M. Inferring the historical patterns of biological evolution. *Nature* **401**, 877–884 (1999).

54. Nagelkerke, N. J. D. A note on a general definition of the coefficient of determination. *Biometrika* **78**, 691–692 (1991)
