# Supplementary material for: Independent origin of large labyrinth size in turtles
Source: Nat Commun. 2022 Oct 11;13:5807. doi: 10.1038/s41467-022-33091-5 (PMC9553989; doi:10.1038/s41467-022-33091-5)
Supplement: Supplementary file 3 — Description of Additional Supplementary Files [file 41467_2022_33091_MOESM3_ESM.pdf]

Description of Additional Supplementary Files

File Name: Supplementary Data 1
Description: Spreadsheet including specimen data, and MorphoSource links to 3D models for each specimen. The CT data are linked to each 3D model within MorphoSource. For datasets with restricted download policies implemented by the museums in charge of the specimens, contact details as listed in MorphoSource are provided.

File Name: Supplementary Data 2
Description: Spreadsheet including specimen data, explanatory variables and measurements for turtle labyrinth analyses. This is read by scripts provided as Datasets 10–13 & 16.

File Name: Supplementary Data 3
Description: Collection of turtle landmark data, as individual csv files. This is read by scripts provided as Datasets 10–30 & 16.

File Name: Supplementary Data 4
Description: Csv file containing information about sliding semilandmarks (for GPA analysis). This is read by scripts provided as Datasets 10–13 & 16.

File Name: Supplementary Data 5
Description: Csv file containing colour codes for landmarks, used for deformation plots along PC axes. This is read by scripts provided as Datasets 10–13 & 16.

File Name: Supplementary Data 6
Description: Csv file containing age data for fossil turtle species, alongside information about the fossil prevenance and museum staff responsible for curation and/or collection management.

File Name: Supplementary Data 7
Description: Text file containing cal3-calibrated phylogenetic tree in nexus syntax. This is read by scripts provided as Datasets 10–13 & 16.

File Name: Supplementary Data 8
Description: Text file containing mbl-calibrated phylogenetic tree in nexus syntax. This is read by scripts provided as Datasets 10–30 & 16.

File Name: Supplementary Data 9
Description: Text file with R script to load landmark data, variable data, phylogenetic data from Datasets 2–5 & 7–8 and to 2B-PLS analysis for te verification of our landmarking scheme.

File Name: Supplementary Data 10
Description: Text file with R script to load landmark data, variable data, phylogenetic data from Datasets 2–5 &7–8 and to perform GPA and PCA analysis. Script creates Figure 1 from main text.

File Name: Supplementary Data 11
Description: Text file with R script to load landmark data, variable data, phylogenetic data from Datasets 2–5 & 7–8 and to perform GPA analysis and labyrinth shape regressions.

File Name: Supplementary Data 12
Description: Text file with R script to perform size- and braincase aspect ratio-corrected PCA and regression analyses, and to get deformation plots. Script creates Figure 2 from main text.

File Name: Supplementary Data 13
Description: Text file with R script to load landmark data, variable data, phylogenetic data from Datasets 2–5 & 7–8 and to perform GPA analysis and labyrinth size regressions and model comparisons.

File Name: Supplementary Data 14
Description: Spreadsheet showing full list of models tested in turtle labyrinth size regressions and model comparison data (AICc, etc.) for analysis runs based on cal3-calibrated phylogenetic tree.

File Name: Supplementary Data 15
Description: . Spreadsheet showing full list of models tested in turtle labyrinth size regressions and model comparison data (AICc, etc.) for analysis runs based on mbl-calibrated phylogenetic tree.

File Name: Supplementary Data 16
Description: Text file with R script to load landmark data, variable data, phylogenetic data from Datasets 2–5 & 7–8 and to perform GPA analysis, labyrinth size regression analysis used for Figure 3 in main text, and ancestral state reconstructions used also in Figure 3.

File Name: Supplementary Data 17
Description: Spreadsheet including specimen data and cranial measurements for amniote labyrinth GPA and labyrinth size plot. This is read by the script provided as Dataset 20.

File Name: Supplementary Data 18
Description: Collection of turtle landmark data, as individual csv files. This is the data file for script provided as Dataset 20.

File Name: Supplementary Data 19
Description: Csv file containing information about sliding semilandmarks (for GPA analysis). This is read by the script provided as Dataset 20.

File Name: Supplementary Data 20
Description: Text file with R script to load amniote landmark data, amniote measurements, and sliders from Datasets 17–19 and to perform GPA analysis and plots shown used as Figure 4 of main text.
