# Supplementary material for: Independent origin of large labyrinth size in turtles
Source: Nat Commun. 2022 Oct 11;13:5807. doi: 10.1038/s41467-022-33091-5 (PMC9553989; doi:10.1038/s41467-022-33091-5)
Supplement: Supplementary file 5 — Reporting Summary [file 41467_2022_33091_MOESM5_ESM.pdf]

nature portfolio

Corresponding author(s): Serjoscha Evers

Last updated by author(s): Aug 24, 2022

Reporting Summary

Nature Portfolio wishes to improve the reproducibility of the work that we publish. This form provides structure for consistency and transparency in reporting. For further information on Nature Portfolio policies, see our Editorial Policies and the Editorial Policy Checklist.

Statistics

For all statistical analyses, confirm that the following items are present in the figure legend, table legend, main text, or Methods section.

n/a	Confirmed	
☐	☒	The exact sample size (*n*) for each experimental group/condition, given as a discrete number and unit of measurement
☐	☒	A statement on whether measurements were taken from distinct samples or whether the same sample was measured repeatedly
☐	☒	The statistical test(s) used AND whether they are one- or two-sided<br>*Only common tests should be described solely by name; describe more complex techniques in the Methods section.*
☐	☒	A description of all covariates tested
☐	☒	A description of any assumptions or corrections, such as tests of normality and adjustment for multiple comparisons
☐	☒	A full description of the statistical parameters including central tendency (e.g. means) or other basic estimates (e.g. regression coefficient) AND variation (e.g. standard deviation) or associated estimates of uncertainty (e.g. confidence intervals)
☐	☒	For null hypothesis testing, the test statistic (e.g. F, t, r) with confidence intervals, effect sizes, degrees of freedom and P value noted<br>*Give P values as exact values whenever suitable.*
☒	☐	For Bayesian analysis, information on the choice of priors and Markov chain Monte Carlo settings
☐	☒	For hierarchical and complex designs, identification of the appropriate level for tests and full reporting of outcomes
☐	☒	Estimates of effect sizes (e.g. Cohen's *d*, Pearson's *r*), indicating how they were calculated

Our web collection on statistics for biologists contains articles on many of the points above.

Software and code

Policy information about availability of computer code

Data collection	The data that underlie this study are CT scans of biological and palaeontological specimens. The specimens are deposited in museum collections and are identified by their collection numbers (supplements includes full list). The CT scans performed for this study were deposited in the online repository MorphoSource, and additional CT scans of other studies were additionally used. All CT scan availability statements are listed in Supplementary Data 1. The 3D labyrinth models used for shape analysis were segmented from these CT scans using the software Mimics v.15-19, and these 3D models were deposited in MorphoSource. The 3D labyrinth models were landmarked in the software Avizo lite 9.2, and landmark data (i.e. 3D coordinate data) are provided in the supplements as csv files.
Data analysis	Analysis of landmark data was carried out in the statistical programming environment R (version 3.6.0) using standard functions of published packages as cited in the methods. The packages used are the following: geomorph v. 3.1.0; paleotree v.3.3.0; Claddis 0.6.3, ape 5.0, qPCR v. 1.4.1; nlme v. 3.1.141. The R code is deposited in Github/Zenodo, and can be accessed via this link: doi.org/10.5281/zenodo.7024572

For manuscripts utilizing custom algorithms or software that are central to the research but not yet described in published literature, software must be made available to editors and reviewers. We strongly encourage code deposition in a community repository (e.g. GitHub). See the Nature Portfolio guidelines for submitting code & software for further information.

Data

Policy information about availability of data

All manuscripts must include a data availability statement. This statement should provide the following information, where applicable:

- Accession codes, unique identifiers, or web links for publicly available datasets
- A description of any restrictions on data availability
- For clinical datasets or third party data, please ensure that the statement adheres to our policy

All CT data gathered for this study were deposited in MorphoSource. All 3D models were deposited in MorphoSource, and can be accessed here: www.morphosource.org/projects/000372533. Supplementary Data 1 list webpage links to the labyrinth models of all used amniote species to facilitate download. The parent CT scans for each model are directly linked with the deposited 3D model in MorphoSource, with a few exceptions when CT data were previously deposited by other authors in different repositories. The CT scan availability for scans not currently housed in MorphoSource, including information about restricted download policies implemented by many museums within MorphoSource, is detailed in Supplementary Data 1. Supplementary Text (including supplementary Tables and Figures) and Supplementary Data files were uploaded directly with the journal. Source data are provided with this paper. Supplementary Data files are also available on GitHub at (https://github.com/SerjoschaEvers/Turtle-Labyrinth-Ecomorphology-and-Evolution-Data), with the version published in this paper available on Zenodo at (doi.org/10.5281/zenodo.7024572)

Field-specific reporting

Please select the one below that is the best fit for your research. If you are not sure, read the appropriate sections before making your selection.

☐ Life sciences ☐ Behavioural & social sciences ☒ Ecological, evolutionary & environmental sciences

For a reference copy of the document with all sections, see nature.com/documents/nr-reporting-summary-flat.pdf

Ecological, evolutionary & environmental sciences study design

All studies must disclose on these points even when the disclosure is negative.

Study description	This study takes 3D landmark data collected from 3D models of endossoeus labyrinths generated from CT scans, and calculates shape variation and labyrinth centroid size from the landmark data using geometric morphometrics. Shape and size data are used as response variables in multivariate phylogenetic regression analyses. We used observable ecological traits and functional parameters of extant turtles as explanatory variables for these analyses. Our regressions test specific hypotheses about whether our explanatory variables explain labyrinth shape and size variation in turtles. Labyrinth size was further optimized across a turtle phylogeny to document patterns of labyrinth size evolution across the turtle tree of life. Turtle labyrinth sizes relative to head size were compared against such data from other amniotes (Benson et al. 2017: birds; Bronzati et al. 2021: archosaurs).
Research sample	The sample consists of individuals for extant and fossil turtle specimens, all of which were collected and accessioned in museum collections prior to our sample. Samping was achieved by visiting these museums and CT scanning materials at local facilities. Extant turtle specimens were selected to maximize the phylogenetic and ecological breadth of the sample ("diversified sampling strategy"), and include species of all major turtle groups (i.e., chelids, pelomedusids, podocnemidids, trionychids, carettochelyids, testudinids, emydids, geoemydids, platytsernids, chelydrids, dermatemydids, kinosternids, dermochelyids, cheloniids). A full list of species with museum identifiers is given in the supplements as Supplementary Data 2. Fossils were selected based on their phylogenetic positions, and included when they represent stem taxa of the entire lineage of Testudines (e.g., Triassic stem turtles, early Jurassic perichelidians, meiolaniformes, paracryptodires, thalassochelydians), or when they represent stem taxa of important modern lineages (e.g., Adocus for Trionychia). Fossils were also selected based on availability of three-dimensionally preserved skull material. Sex and exact ontogenetic age were unknown for fossils and extant fossils, but sampled individuals predominantly were adult individuals. Some species were represented by several specimens of different sizes, and these support low intraspecific variability. Intraspecific variation (sexual, ontogenetic) was thus taken to be lower than the interspecific variation between taxa that was the primary target of this study. As such, each sample is meant to represent an evolutionary lineages (not a population, etc.). Each sample was CT scanned, and the left endosseous labyrinth was digitally segmented from the scan. For some specimens, right labyrinths were reconstructed when the left side was damaged (particularly fossils).
Sampling strategy	The sampling of extant turtles follows a "diversified sampling" in which members of all major turtle clades are sampled. Fossil turtles were added according to fossil availability and preservation, but a specific sampling focus was on fossils belonging to the stem lineage of turtles and those fossils that belong to clades that document independent ecological transitions during the evolutionary history of turtles.
Data collection	All authors contributed to CT scanning specimens used in this study. Serjoscha Evers segmented all turtle labyrinth models and did all the landmarking for all turtle specimens. Non-turtle specimens were taken from Benson et al. (2017; birds), Bronzati et al. (2021; non-bird archosaurs). These studies used the same landmarking scheme (partially also done by Serjoscha Evers for the previous studies) so that these data were directly compatible. Catherine Johnson segmented and landmarked mammal specimens, the labyrinth models and CT data for which were deposited in MorphoSource.
Timing and spatial scale	Our sample covers the entire evolutionary history of shelled turtles, with samples from c. 230 Ma until the present (extant turtles). The exact ages of the 53 fossil species used in this study are detailed in Supplementary Data 6. The geographic origin of the fossils is specified in Supplementary Data 6, too. It is difficult to specify the exact spatial scale, because the fossils come from different geological ages during which continents had a different position. However, fossils include specimens from what today is Afrika, Asia, Australia, Europe, North America, South America. The extant turtle samples is also global, with species from all continents (but

	Antartica) included.
Data exclusions	No data were specifically excluded for this study. However, the nature of our analyses don't allways allow the inclusion of fossil data. For multivariate phylogenetic regressions, ecological data must be available, so that only extant turtles could be analysed in this way (excluding fossils). Also, when multiple specimens of one species were available, phylogenetic regressions only allow inclusion of one specimen, in which case the largest, ontogenetically most mature specimen was chosen.
Reproducibility	Anatomical data were collected objectively at high precision using micro-CT scanning, and 3D models were segmented from the resulting CT scans. CT scans could be reproduced as all specimens are housed in public museum collections, with specimen details listed in Supplementary Data 1 and 2. 3D models can be re-segmented from the CT data, which are available via MorphoSource. Supplementary Data 2 provides links to specimens on MorphoSource, and also details curatorial/collection management email contacts for those specimens that were deposited under a restricted download policy (following museum instructions). We landmarked 3D models using uniquely relocatable points and algorithmic sampling procedures. These can be replicated using the 3D models deposited at MorphoSource, and R code deposited in Github/Zenodo (doi.org/10.5281/zenodo.7024572). In addition, we deposit all landmark data as Supplementary Data, so all statistical analyses can be reproduced using our exact landmark dataset. We also deposit the code for this procedure, so that reproducing our analyses in exactly the same way with the same dataset is possible and easy. We ran our phylogenetic regression models on separate phylogenies to test the robustness of our results against the effects of different phylogenetic calibrations. All analytical procedures were replicated twice before depositing code on Github to avoid errors. Figure contents can be reproduced step-by-step using the code at Github/Zenodo, but numerical raw data for plots in figures are additionally provided as a Source Data file, also deposited as Supplementary Data 21 (doi.org/10.5281/zenodo.7018452).
Randomization	Our data are species data and thus statistically not independent due to shared evolutionary history. We account for this by using phylogenetic comparative methods, for instance phylogenetic rather than non-phylogenetic regressions. The phylogenetic position of turtles used in this study follow molecular consensus topologies. Ecologies were assigned based on literature reports. No other grouping strategy was used for this work.
Blinding	Blinding in the sense of a clinical study was not relevant because our study objects were dead turtles, some of which have been dead for more than 200 million years. Group assignments of turtles are dictated by published records - for example, for the phylogenetic identity of turtles we follow molecular consensus topologies for turtles, and for ecological group assignments we follow published records of the turtle species' ecologies. Statistical regression models used in our studies establish significances by permutating algorithms (described in the literature developing these methods), which randomly re-assign species to different groups t establish data distributions that can be compared against the analysis using 'true' group assignments.

Did the study involve field work? ☐ Yes ☒ No

Reporting for specific materials, systems and methods

We require information from authors about some types of materials, experimental systems and methods used in many studies. Here, indicate whether each material, system or method listed is relevant to your study. If you are not sure if a list item applies to your research, read the appropriate section before selecting a response.

Materials & experimental systems

n/a	Involved in the study	
☒	☐	Antibodies
☒	☐	Eukaryotic cell lines
☐	☒	Palaeontology and archaeology
☒	☐	Animals and other organisms
☒	☐	Human research participants
☒	☐	Clinical data
☒	☐	Dual use research of concern

Methods

n/a	Involved in the study	
☒	☐	ChIP-seq
☒	☐	Flow cytometry
☒	☐	MRI-based neuroimaging

Palaeontology and Archaeology

Specimen provenance	We did not collect any fossil material ourselves for this study and instead used fossils deposited in public museum repositories. This study thus did not require to obtain specific permits other than curatorial permissions to study specimens, which were made via official email request prior to studying any material. Supplementary Data 6 includes contact emails for curatorial/collection management staff for all fossil turtles sampled for this study. All specimens are registered with various museums that are globally distributed (see Supplementary Data 6 for details). The respective specimens were collected and accessioned historically, prior to our study, and are held legally by their respective institutions, who can be contacted for further information on the specimens. All specimens are listed with their museum identifiers in the supplements, and a list of museum abbreviations is provdied as a key in the supplementary text. We also listed the provenance data for each fossil specimen in Supplementary Data 6 as listed on museum labels and/or first published records of the specimens in question. Specimens were CT scanned with the permissions of the respective curatorial staff at the respective museums. We collected data from nearly fifty museums, a full list of which is provided in the supplements.
Specimen deposition	As we collected no new fossil material, there are no additional specimens deposited because of our study. However, we deposited CT scans of all specimens scanned by ourselves at the online repository MorphoSource, or, in a few cases (see supplements for details on each specimen) with the museum holding the specimens themselves (when we did not get curatorial permission to deposit these data in an online repository). All 3D files are depoisted on MorphoSource.
Dating methods	No new dates are provided, and we used previously reported dates for fossils, which are disclosed in the supplements.

☒ Tick this box to confirm that the raw and calibrated dates are available in the paper or in Supplementary Information.

Ethics oversight	When handling with museum specimens, we followed the respective guidance given by the curatorial team of the museum. However, no ethical guidance was received for this study, particularly as there is not yet a well-established community-standard of fossil ethics. We did not include any private fossil material, as this is often perceived as unethical due to reproducibility issues.

Note that full information on the approval of the study protocol must also be provided in the manuscript.
